# Neural mechanisms of contextual modulation in the retinal direction selective circuit

**DOI:** 10.1038/s41467-019-10268-z

**Published:** 2019-06-03

**Authors:** Xiaolin Huang, Melissa Rangel, Kevin L. Briggman, Wei Wei

**Affiliations:** 1 Department of Neurobiology, The University of Chicago, Chicago, IL 60637 USA; 2 The Committee on Neurobiology Graduate Program, The University of Chicago, Chicago, IL 60637 USA; 3 Department of Computational Neuroethology, Center of Advanced European Studies and Research (caesar), Bonn, 53175 Germany

**Keywords:** Neural circuits, Retina

## Abstract

Contextual modulation of neuronal responses by surrounding environments is a fundamental attribute of sensory processing. In the mammalian retina, responses of On–Off direction selective ganglion cells (DSGCs) are modulated by motion contexts. However, the underlying mechanisms are unknown. Here, we show that posterior-preferring DSGCs (pDSGCs) are sensitive to discontinuities of moving contours owing to contextually modulated cholinergic excitation from starburst amacrine cells (SACs). Using a combination of synapse-specific genetic manipulations, patch clamp electrophysiology and connectomic analysis, we identified distinct circuit motifs upstream of On and Off SACs that are required for the contextual modulation of pDSGC activity for bright and dark contrasts. Furthermore, our results reveal a class of wide-field amacrine cells (WACs) with straight, unbranching dendrites that function as “continuity detectors” of moving contours. Therefore, divergent circuit motifs in the On and Off pathways extend the information encoding of On-Off DSGCs beyond their direction selectivity during complex stimuli.

## Abstract (web summary)

The mechanisms of contextual modulation in direction selective ganglion cells in the retina remain unclear. Here, the authors find that that On-Off direction-selective ganglion cells are differentially sensitive to discontinuities of dark and bright moving edges in the visual environment and, using synapse-specific genetic manipulations with functional measurements, reveal the microcircuits underlying this contextual sensitivity.

## Introduction

The visual information an animal obtains at a local region is profoundly shaped by the surrounding environment. This perceptual attribute has been linked to context-dependent modulation of visual neuronal responses: the activity of a neuron driven by a target stimulus in its classic receptive field (RF) (referred to as RF center) is differentially facilitated or suppressed based on the luminance, contrast and spatiotemporal properties of the stimuli in the surround^1^. Contextual modulation arises early in the visual pathway and is readily detectable in multiple retinal ganglion cell types in the vertebrate retina^2–6^.

On-Off DSGCs, which are well-studied for their encoding of motion direction, are subject to contextual modulation. Sensitivity to motion contexts has been reported in rabbit DSGCs using preferred-direction drifting gratings in the center and surround that differ in spatial phase, spatial frequency or temporal frequency^7^. However, whether On-Off DSGCs prefer discontinuities in spatial texture in the absence of motion is unclear. Furthermore, the contextual modulation of the On and Off responses of DSGCs has not been separately examined. And importantly, the synaptic mechanisms underlying the contextual modulation of On-Off DSGCs are unknown.

**Fig. 1:**
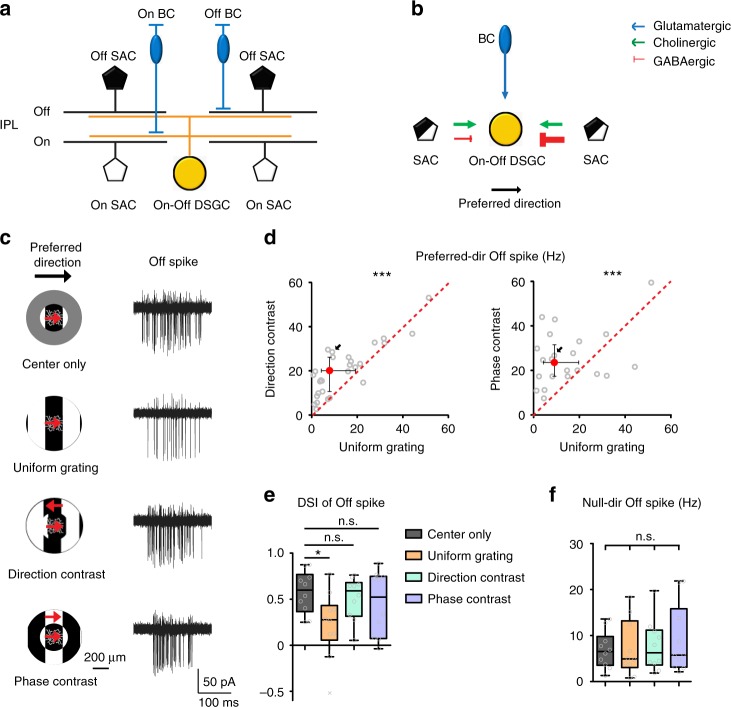
pDSGC Off responses are contextually modulated. **a** Schematic shows side view of the laminar organization of bipolar cells (BCs), SACs and On-Off DSGCs in the inner plexiform layer (IPL). **b** Simplified schematic shows major types of synaptic inputs onto On-Off DSGCs. **c** Left: Schematics show the visual stimulus conditions. A schematic of a pDSGC is shown in the center of each stimulus to indicate the relative sizes of DSGC dendritic arbors and visual stimuli. The red arrows indicate the moving directions of the drifting gratings, and the black arrow at the top indicates the preferred direction of the pDSGC. Right: Example Off spike traces of a pDSGC from a control mouse evoked by the stimuli on the left. Spike traces are overlays of 10 grating cycles (also see Methods) for this and subsequent figures. **d** Scatter plots compare Off firing rate during uniform versus compound gratings. Direction-contrast vs uniform grating: *n* = 29 cells from 13 mice; phase-contrast vs uniform grating: *n* = 22 cells from 9 mice. Wilcoxon signed-rank test, ***p < 0.001. Grey dots represent individual cells, red dots with error bars indicate the median ± IQR, and diagonal red dashed lines are unity lines. The data point corresponding to the example traces was labelled by the black arrow. **e** Direction selectivity index (DSI) of pDSGC Off spiking in different stimulus contexts. Friedman test: *n* = 10 cells from 4 mice, *p = 0.025. Wilcoxon signed-rank test with Bonferroni correction: center only vs uniform grating: *p = 0.0039; center only vs direction-contrast: *p* = 0.23; center only vs phase-contrast: *p* = 0.70. Box plots represent minimum/first quartile/median/third quartile/maximum values, open grey dots represent individual cells, grey crosses are outliers defined by Tukey’s fences. **f** pDSGC Off firing rate when center gratings drift in the null direction. Friedman test: *n* = 10 cells from 4 mice, *p* = 0.29. See also Supplementary Fig. 1 for further identification of Off firing rate during different stimulus contexts, and Supplementary Movie 1 for visual stimuli

A mechanistic understanding of the contextual tuning of On–Off DSGCs requires investigation of the underlying synaptic circuitry. In accordance with the general rule of retinal circuit organization, the responses of DSGCs to bright and dark moving edges are separately processed by the On and Off pathways. The bistratified dendritic arbors of On–Off DSGCs are located in the On and Off sublaminae of the inner plexiform layer that are specialized for processing bright and dark contrasts. At each sublamina, the On or Off dendritic layer receives glutamatergic input from On or Off bipolar cells and lateral cholinergic and GABAergic inputs from On or Off starburst amacrine cells (SACs) (Fig. 1a, b)^8,9^. GABAergic inputs from On and Off SACs to DSGCs are strongest during motion in the null direction and weakest in the preferred direction (Fig. 1b)^10–14^. This directionally tuned inhibition, together with a rich set of other circuit and intrinsic mechanisms, form the neural basis of retinal direction selectivity^15^. Despite an increasingly detailed wiring diagram for the direction selectivity of On-Off DSGCs, contextual modulation of On-Off DSGCs by motion stimuli in the RF surround eludes the current circuit model.

In this study, we determine how the On and Off spiking responses of mouse posterior-preferring On-Off DSGCs (pDSGCs) are differentially modulated by motion contexts and identified the underlying microcircuit motifs in the On and Off pathways. We found that SAC-mediate cholinergic outputs at On and Off sublamina are differentially modulated by a class of WACs with long, straight dendrites that are sensitive to continuity of moving contours. These WACs confer contextual sensitivity of pDSGC responses by shaping SAC-mediated cholinergic excitation. Our results link the existing circuit motifs mediating direction selectivity to the extensive inner retinal network, highlighting a multilayered circuit architecture that dynamically engages specific microcircuit motifs to process motion information according to the composition of visual features in the visual stimulus.

## Results

### pDSGC Off responses are sensitive to motion discontinuities

To determine how On and Off responses of On–Off DSGCs to motion stimuli in the RF center are modulated by motion patterns in the surround, we performed loose-patch recordings from a subtype of On–Off DSGCs that prefer motion in the posterior direction (pDSGCs), which are labeled by the Drd4-GFP transgenic mouse line^16^. Full-field motion stimuli were divided into center and surround areas where drifting square-wave gratings with the same spatial and temporal frequency were presented (see Methods). The center gratings always moved in the preferred direction of the pDSGCs, while the surround gratings differed in direction or spatial phase to create direction-contrast or phase-contrast between center and surround regions (Fig. 1c, schematics; Supplementary Movie 1). We refer to both direction-contrast and phase-contrast gratings as compound gratings. Off and On responses of pDSGCs to these drifting gratings showed comparable spike firing rates, and were well separated as dark and bright edges of the gratings traveled across the cell’s RF (Supplementary Fig. 1a, d). We examined the Off and On responses separately for their contextual modulation.

pDSGC Off responses in the preferred direction were maximal when the drifting grating was confined to the RF center, and decreased by the inclusion of stimuli in the surround during uniform and compound grating stimulation (Supplementary Fig. 1b). Moreover, surround gratings alone did not evoke spiking of pDSGCs regardless of motion directions (Supplementary Fig. 1d, e), consistent with the presence of a suppressive surround in these cells^17^. Although gratings in the surround suppressed the center response, this surround suppression was significantly stronger for uniform grating than compound gratings (Fig. 1c, d), suggesting that the Off response is sensitive to motion contexts.

We next tested the impact of stimulus contexts on the direction selectivity of pDSGCs by calculating direction selectivity index (DSI, see Methods) of their spiking activity evoked by uniform and compound grating stimuli. Compared to center-only condition, DSI was unaffected by the inclusion of surround gratings with direction-contrast or phase-contrast, but was significantly reduced during uniform grating (Fig. 1e). This reduction was due to the stronger surround suppression of preferred-direction response during uniform grating, since the null-direction spike counts remained low and were comparable under all conditions (Fig. 1f). Therefore, compared to continuous moving edges, discontinuities of contours between center and surround improve the direction selectivity of pDSGC Off response by enhancing the preferred-direction spiking.

### Cholinergic excitation of pDSGCs is contextually sensitive

**Fig. 2:**
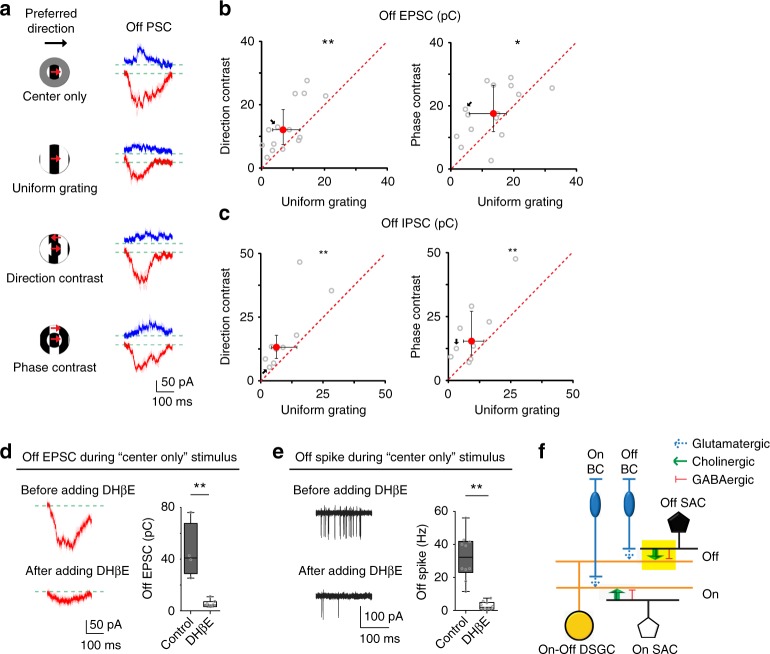
Contextually sensitive cholinergic excitation underlying the differential modulation of pDSGC Off response. **a** Whole-cell recording traces of Off IPSCs (blue) and Off EPSCs (red) from a pDSGC in a control mouse. PSC traces represent trial average (darker traces) and SEM (lighter traces) for this and subsequent figures. **b** Scatter plots compare Off EPSC charge transfer between uniform grating and compound gratings. Direction-contrast vs uniform grating: *n* = 15 cells from 11 mice, **p = 0.0043; Phase-contrast vs uniform grating: *n* = 15 cells from 9 mice, *p = 0.035 (Wilcoxon signed-rank test). **c** Same as **b**, but for Off IPSC charge transfer. Direction-contrast vs uniform grating: *n* = 9 cells from 5 mice, **p = 0.0039; Phase-contrast vs uniform grating: *n* = 10 cells from 6 mice, **p = 0.0098. **d** Left: Example Off EPSC traces of a pDSGC during center only grating before and after adding DHβE. Right: Summary graph of pDSGC Off excitatory charge transfer in control (Ames’ solution, *n* = 4 cells from 2 mice) or in the presence of DHβE (*n* = 7 cells from 2 mice): **p = 0.0061 (Wilcoxon rank-sum test). See Supplementary Fig. 2a for Off IPSC as a negative control. **e** Left: Example Off spike traces of a pDSGC during center only grating before and after adding DHβE. Right: Summary graph of pDSGC Off spike firing rate before (in Ames’ solution) or after adding DHβE: *n* = 10 cells from 6 mice, **p = 0.0020 (Wilcoxon signed-rank test). **f** Schematic shows the synaptic connections modulated by motion context in the Off pathway (yellow filled rectangle). Glutamatergic BC inputs are shown in dotted arrows indicating their contribution is little under the experimental conditions in this study. See also Supplementary Fig. 2c for Off EPSC charge transfer under different stimulus contexts in the present of DHβE

The stronger surround suppression of the Off spiking response by uniform grating may result from stronger suppression of excitatory inputs or stronger enhancement of inhibitory inputs onto pDSGCs. To understand the synaptic mechanism underlying this differential suppression effect, we measured the excitatory and inhibitory postsynaptic currents (EPSCs and IPSCs) of pDSGCs using whole-cell voltage clamp recording. We separated the postsynaptic currents into On and Off components based on the temporal windows during which the pDSGCs were activated by bright and dark phases of the square-wave gratings (see Methods). Consistent with the spiking response, the Off component of EPSCs showed stronger surround suppression by uniform grating (Fig. 2a, b). In addition to EPSCs, the Off component of IPSCs of pDSGCs also showed stronger surround suppression by uniform grating (Fig. 2c), indicating that the suppressive mechanism affects both excitatory and inhibitory inputs of pDSGCs. However, stronger surround suppression of pDSGC spiking activity by uniform grating indicates that the effect of reduced inhibition is not sufficient to offset the effect of reduced excitation. We reason that excitation onto pDSGCs plays a dominant role in shaping the spiking response when motion is in the preferred direction. In contrast to the strong excitatory drive, the directionally tuned inhibition is the weakest and the most delayed in this direction^14^, and therefore contribute little to pDSGC spiking generation. In support of this hypothesis, we found a strong correlation between EPSC waveform and spiking activity in the preferred direction (*r* = 0.77±0.03 for center-only stimulus, *n* = 6 cells). Therefore, contextually sensitive Off EPSCs are responsible for the stronger surround suppression of the pDSGC Off response by uniform grating.

Excitation onto pDSGCs consists of glutamatergic inputs from bipolar cells and cholinergic inputs from SACs. Previous studies using moving spots have shown that the relative contributions of these two types of excitation depend on stimulus conditions^18,19^. We thus examined the relative contributions of glutamatergic and cholinergic components to pDSGC EPSCs measured under our visual stimulus conditions. Since we observed concomitant contextual modulation of EPSCs and IPSCs of pDSGCs, this suggests that the modulatory mechanism may target SACs to simultaneously reduce their acetylcholine and GABA release onto pDSGCs. To test whether cholinergic input is the predominant mode of excitation during the drifting grating stimuli used in our study, we applied the nicotinic acetylcholine receptor antagonist DHβE to selectively block the cholinergic component of EPSCs^20,21^. Both On and Off components of EPSCs during center-only gratings were dramatically reduced in the presence of DHβE (Fig. 2d, Supplementary Fig. 2b), indicating that the main component of pDSGC EPSCs was cholinergic. Consistent with the significantly reduced EPSCs, pDSGC spiking responses were largely eliminated after adding DHβE (Fig. 2e, Supplementary Fig. 2b). We didn’t detect a statistically significant contextual modulation in the residual weak EPSCs (Supplementary Fig. 2c, d). The spiking data were not analyzed since there were barely any spikes for quantification during full-field gratings in the presence of DHβE. Therefore, the differential surround suppression of pDSGC Off spiking by continuous and discontinuous drifting gratings in this study is primarily due to the modulation of the cholinergic inputs from Off SACs to pDSGCs (Fig. 2f).

### Direct GABAergic inhibition from WACs onto Off SACs

**Fig. 3:**
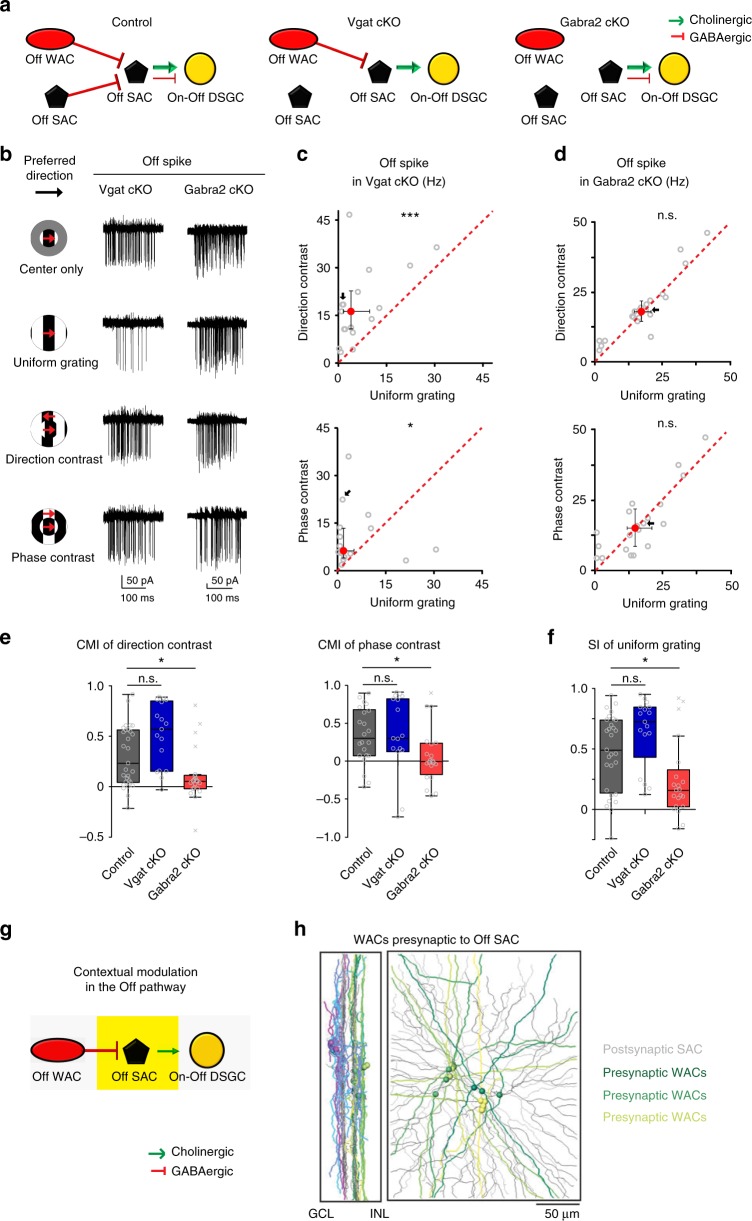
Contextual modulation of pDSGC Off response is mediated by direct inhibition onto SACs. **a** Schematic diagrams show the GABAergic synaptic connections in control, Vgat cKO and Gabra2 cKO mice. **b** Example pDSGC Off spike traces from a Vgat cKO mouse and a Gabra2 cKO mouse. **c** Scatter plots compare pDSGC Off firing rate in Vgat cKO mice during uniform grating and compound gratings. Direction-contrast vs uniform grating: *n* = 17 cells from 11 mice, ***p < 0.001; Phase-contrast vs uniform grating: *n* = 16 cells from 10 mice, *p = 0.039 (Wilcoxon signed-rank test). **d** Same as **c**, but for Gabra2 cKO mice. Direction-contrast vs uniform grating: *n* = 21 cells from 9 mice, *p* = 0.065; Phase-contrast vs uniform grating: *n* = 20 cells from 9 mice, p = 0.88. **e**, Summary graphs compare CMI of pDSGC Off response in control, Vgat cKO and Gabra2 cKO mice. CMI of direction-contrast: Kruskal–Wallis test, ***p = 0.0004. Wilcoxon rank-sum test with Bonferroni correction: Control vs Vgat cKO: *p* = 0.028; Control vs Gabra2 cKO: *p = 0.019. CMI of phase-contrast, Kruskal–Wallis test: *p = 0.024. Wilcoxon rank-sum test with Bonferroni correction: Control vs Vgat cKO: *p* = 0.40; Control vs Gabra2 cKO: *p = 0.015. **f** Summary graph compares SI of pDSGC Off firing rate during uniform grating stimulus between control, Vgat cKO and Gabra2 cKO mice. Kruskal-Wallis test: **p = 0.0012. Wilcoxon rank-sum test with Bonferroni correction: Control vs Vgat cKO: *p* = 0.041; Control vs Gabra2 cKO: *p = 0.020. **g** Schematic shows the synaptic connections involved in contextual modulation in the Off pathway of the direction selective circuit (yellow filled rectangle). **h** Connectomic reconstruction of wide-field amacrine cells (WACs) presynaptic to Off SACs. Off SACs are colored in grey, while Off WACs presynaptic to Off SACs are colored in green, yellow-green and yellow. Balls represent somas. (Modified from Ding et al.^23^, Extended Fig. 4d). See also Supplementary Fig. 4 for Off EPSC of pDSGCs in Vgat cKO and Gabra2 cKO mice

What mediates the contextually sensitive surround suppression of the cholinergic inputs from Off SACs to pDSGCs? One candidate is the GABAergic inhibition from neighboring SACs^22^, since reciprocal inhibitory inputs between neighboring SACs are the most numerous among the total GABAergic inputs onto SACs^23^. In addition to the reciprocal SAC – SAC inhibition, Off SACs also receive GABAergic inhibition from non-starburst amacrine cells^24^, which are identified to be WACs based on connectomic tracing^23^ (Fig. 3a, Control). To determine the synaptic locus that mediates the differential surround suppression, we applied a loss-of-function approach using two types of SAC-targeted genetic manipulations. The first one perturbs GABA release from the SAC by conditionally knocking out the vesicular GABA transporter (Vgat) gene in SACs. In these Vgat cKO mice, both SAC – DSGC and SAC – SAC inhibition are disrupted with no detectable developmental compensation in the rest of the direction selective circuit (Fig. 3a, Vgat cKO)^20,24^. As previously reported^20^, IPSCs of pDSGCs are strongly reduced in Vgat cKO mice and the residual inhibition is non-directional (Supplementary Fig. 3). In the other conditional knock-out mouse line, all direct GABAergic inhibition onto SACs, including SAC – SAC mutual inhibition and WAC – SAC inhibition, is abolished by selectively removing the α2 subunit of GABA_A_ receptors (Gabra2) from SACs (Fig. 3a, Gabra2 cKO)^24–26^.

In Vgat cKO mice where GABA release from SACs was disrupted, the contextual sensitivity of pDSGC Off response was not affected. We found both the Off spiking and Off EPSCs were still more strongly suppressed by uniform grating than by compound gratings (Fig. 3b, c for spiking, Supplementary Fig. 4a, b for EPSCs). To quantify the level of contextual modulation, a contextual modulation index (CMI) of pDSGC spiking is calculated as (N_compound grating_ − N_uniform grating_)/(N_compound grating_ + N_uniform grating_), where N is the spike count. For both direction-contrast and phase-contrast gratings, the CMI of pDSGC Off spiking in Vgat cKO mice remained unchanged compared to that in control mice (Fig. 3e). The normal contextual tuning of the Off response in Vgat cKO mice indicates that GABA release from SACs is not required in this process. This result rules out the involvement of two synaptic loci. First, synaptic inhibition onto pDSGCs is not necessary for contextual modulation, since normal contextual sensitivity of pDSGCs persists in the absence of SAC – DSGC inhibition despite its critical role in direction selectivity. This lends further support to the important role of synaptic excitation onto pDSGCs in contextual modulation as demonstrated in control mice (Fig. 2). Second, the reciprocal inhibition between Off SACs is not required for the stronger surround suppression of pDSGC Off response by uniform grating, excluding the possibility that Off SACs are inhibited by neighboring SACs to reduce their acetylcholine release onto pDSGCs.

We next tested whether the GABAergic inhibition from WACs to SACs plays a role in the contextual modulation. In Gabra2 cKO mice, both the Off spiking and Off EPSCs of pDSGCs were similar during uniform grating and compound gratings (Fig. 3b–d for spiking, Supplementary Fig. 4a, c for EPSCs). The CMI of pDSGC Off spiking in Gabra2 cKO mice was significantly reduced compared to that in control mice (Fig. 3e). The disruption of Off contextual modulation in Gabra2 cKO mice was caused by diminished surround suppression during uniform grating (Fig. 3f). Since we have already ruled out the contribution of SAC – SAC mutual inhibition to Off contextual modulation, the reduced contextual sensitivity of pDSGCs in Gabra2 cKO mice is attributed to the feedforward inhibition from Off WACs to Off SACs (Fig. 3g). Indeed, Off WACs presynaptic to Off SACs have been reconstructed from a connectomic study using serial block face electron microscopy (SBEM)^27^ (Fig. 3h)^23^.

Long-range inhibition from the surround often involves spiking WACs^28–33^. Therefore, we tested whether the contextual modulation of Off SAC acetylcholine release depends on spiking activity. When the voltage-gated Na^+^ channel antagonist tetrodotoxin (TTX) was applied to the control retina, the stronger suppression of pDSGC Off EPSCs during uniform gratings was completely abolished (Supplementary Fig. 5a, b). This result indicates a prominent involvement of spiking amacrine cells in pDSGC surround suppression either by directly inhibiting Off SACs or acting upstream of the WAC – Off SAC synapse. However, we cannot rule out a role of spiking activity from bipolar cells in this process. Considering TTX affects the activity of multiple cell types in the retina^34–36^, the relevant neural circuits underlying this TTX-mediated effect await further studies.

### Weak contextual modulation of pDSGC On responses

**Fig. 4:**
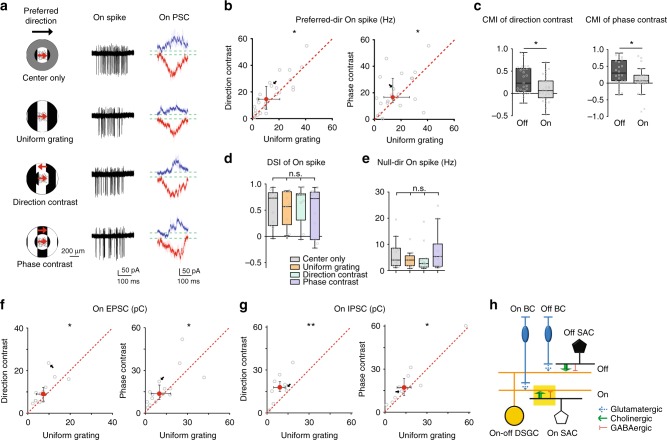
Contextually sensitive cholinergic excitation underlies the weak contextual modulation of pDSGC On response. **a** Left: Visual stimuli with different motion contexts. Middle: Example On spike traces of a pDSGC from a control mouse. Right: Whole-cell recording traces of On IPSCs (blue) and On EPSCs (red) from a pDSGC in a control mouse. **b** Scatter plots compare pDSGC preferred-direction On spike firing rate during uniform grating and compound gratings. Direction-contrast vs uniform grating: n = 27 cells from 10 mice, *p = 0.036; Phase-contrast vs uniform grating: *n* = 22 cells from 8 mice, *p = 0.046 (Wilcoxon signed-rank test). **c** Summary graphs compare CMI of pDSGC Off and On response in control mice. CMI of direction-contrast: *p = 0.014; CMI of phase-contrast: *p = 0.034; Wilcoxon rank-sum test, using the same sample group as Figs 1d and 3b. **d** DSI of pDSGC On response under different stimulus conditions. Friedman test: *n* = 11 cells from 4 mice, *p* = 0.48. **e** pDSGC On firing rate when center gratings drift in the null direction. Friedman test: *n* = 11 cells from 4 mice, *p* = 0.43. **f** Same as **b**, but for On EPSC charge transfer. Direction-contrast vs uniform grating: *n* = 13 cells from 10 mice, *p = 0.017; Phase-contrast vs uniform grating: *n* = 15 cells from 9 mice, *p = 0.035. **g** Same as **b**, but for On IPSC charge transfer. Direction-contrast vs uniform grating: *n* = 9 cells from 5 mice, **p = 0.0039; Phase-contrast vs uniform grating: *n* = 10 cells from 6 mice, *p = 0.037. **h** Schematic diagram shows the synaptic connections modulated by motion context in the On pathway (yellow filled rectangle). See also Supplementary Fig. 1a showing comparable spike firing rate of pDSGC On and Off response, Supplementary Fig. 1c for suppression index of On spikes and Supplementary Fig. 2b for pDSGC On responses in the present of DHβE

The preferred-direction On spiking response of pDSGC also showed contextual sensitivity (Fig. 4a,b; Supplementary Fig. 1c). However, the CMI of On responses was significantly lower than that of Off responses for both direction-contrast and phase-contrast gratings (Fig. 4c), indicating that the On responses during uniform and compound gratings are less different. Because of the moderate contextual modulation in the On pathway, the DSI of On responses was not significantly different across stimulus conditions (Fig. 4d). The null-direction On spiking remained weak and did not show contextual sensitivity (Fig. 4e).

Similar to the Off pathway, the stronger surround suppression of pDSGC On spiking during uniform grating was due to weaker On EPSCs (Fig. 4f), but not enhanced On IPSCs (Fig. 4g). Since the main component of pDSGC On EPSCs is cholinergic (Supplementary Fig. 2b), the contextual modulation of pDSGC On response was a result of contextually sensitive acetylcholine release from On SACs (Fig. 4h, Supplementary Fig. 2d).

### Contextual modulation of bipolar cell (BC) – On SAC synapse

**Fig. 5:**
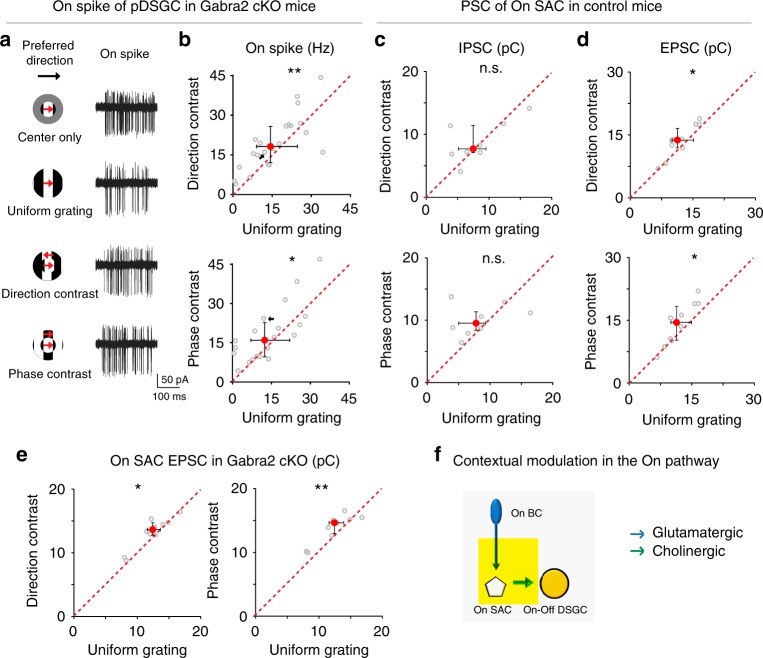
The contextual sensitivity of pDSGCs and On SACs is mediated by the excitatory inputs but not inhibitory inputs onto On SACs. **a** On spike traces of an example pDSGC from a Gabra2 cKO mouse under different stimulus conditions. See also Supplementary Fig. 6a for example EPSC traces from a pDSGC in Gabra2 cKO mouse. **b** Scatter plots compare pDSGC On spike firing rate during uniform grating versus compound gratings in Gabra2 cKO mice. Direction-contrast vs uniform grating: *n* = 22 cells from 12 mice, **p = 0.0094; Phase-contrast vs uniform grating: *n* = 20 cells from 12 mice, *p = 0.048. Wilcoxon signed-rank tests were used in this and subsequent scatter plots. See also Supplementary Fig. 6b for comparison of On EPSC charge transfer of pDSGCs in Gabra2 cKO mice. **c** Scatter plots of On SAC inhibitory charge transfer in control mice, n = 8 cells from 4 mice. Direction-contrast vs uniform grating: p = 0.95; Phase-contrast vs uniform grating: *p* = 0.15. **d** Scatter plots of On SAC excitatory charge transfer in control mice, *n* = 10 cells from 4 mice. Direction-contrast vs uniform grating: *p = 0.020; Phase-contrast vs uniform grating: *p = 0.027. **e** Scatter plots of On SAC excitatory charge transfer in Gabra2 KO mice, *n* = 10 cells from 2 mice. Direction-contrast vs uniform grating: *p = 0.037; Phase-contrast vs uniform grating: **p = 0.0098. **f** Schematic diagram shows the acetylcholine release of On SAC is contextually modulated by On BC – On SAC excitatory inputs

Since contextually sensitive cholinergic excitation of pDSGCs from SACs underlies the stronger surround suppression of pDSGC spiking by uniform gratings in both the On and Off pathways, we next tested whether the On pathway also relies on direct inhibition of On SACs for contextual modulation. In sharp contrast to the Off response, the contextual sensitivity of pDSGC On spiking and On EPSCs is not affected by conditionally knocking out Gabra2 from SACs, and both of them remained weakly contextually sensitive like those in control mice (Fig. 5a, b for spiking, Supplementary Fig. 6 for EPSCs). Therefore, direct inhibition onto On SACs is not required for the contextual modulation in the On pathway. To further rule out a role of direct inhibitory inputs onto On SACs, we examined IPSCs of On SACs during drifting grating stimuli (Fig. 5c). We did not detect stronger On SAC IPSCs during uniform grating, indicating that stronger suppression of acetylcholine release from On SACs by uniform grating is not due to stronger inhibitory inputs onto On SACs.

Another candidate synaptic locus for the contextual modulation of On SACs is bipolar cell terminals presynaptic to On SACs. To test if bipolar cell inputs onto On SACs are more suppressed by uniform grating than compound gratings, we performed whole-cell recordings from On SACs and measured the charge transfer of EPSCs during drifting grating stimuli. We found that EPSCs of On SACs were more suppressed by uniform grating in both control (Fig. 5d) and Gabra2 cKO mice (Fig. 5e). These results indicate that the contextual modulation in the On pathway depends on contextually modulated glutamate release of bipolar cells onto On SACs (Fig. 5f). Therefore, distinct microcircuits are involved in the contextual modulation in the On and Off pathways (Figs. 3g and 5f).

### Enhanced contextual sensitivity of On pathway in Vgat cKOs

**Fig. 6:**
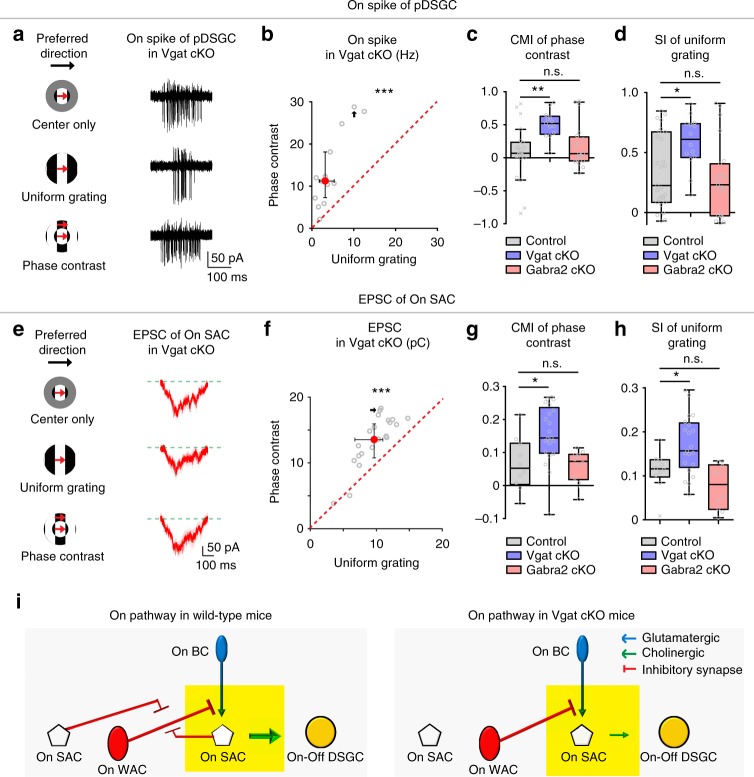
Conditionally knocking out Vgat in SACs unmasks strong contextual modulation in the pDSGC On pathway. **a** Example On spike traces of a pDSGC in Vgat cKO mouse. **b** Scatter plot compares pDSGC On firing rate during uniform grating and phase-contrast grating in Vgat cKO mice: *n* = 13 cells from 7 mice, ***p < 0.001. **c** Summary graph compares CMI of phase-contrast for pDSGC On response in control, Vgat cKO and Gabra2 cKO mice: Kruskal–Wallis test: **p = 0.0016. Wilcoxon rank-sum test with Bonferroni correction: Control vs Vgat cKO: **p = 0.00080; Control vs Gabra2 cKO: *p* = 0.78. **d** Summary graph compares SI of pDSGC On firing rate during uniform grating between control, Vgat cKO and Gabra2 cKO mice: Kruskal-Wallis test: **p = 0.0084. Wilcoxon rank-sum test with Bonferroni correction Control vs Vgat cKO: *p = 0.014; Control vs Gabra2 cKO: *p* = 0.53. **e** EPSC traces of an example On SAC in Vgat cKO mouse. **f** Scatter plot of On SAC excitatory charge transfer during uniform grating versus phase-contrast in Vgat cKO mice: *n* = 21 cells from 5 mice, ***p < 0.001. See also Supplementary Fig. 7 for comparison of On SAC IPSC charge transfer in Vgat cKO mice. **g** Summary graph compares CMI of phase-contrast for On SAC excitatory charge transfer in control, Vgat cKO and Gabra2 cKO mice: Kruskal-Wallis test: **p = 0.0015. Wilcoxon rank-sum test with Bonferroni correction: Control vs Vgat cKO: *p = 0.0083; Control vs Gabra2 cKO: *p* = 0.91. **h** Summary graph compares SI of On SAC excitatory charge transfer during uniform grating between control, Vgat cKO and Gabra2 cKO mice: Kruskal–Wallis test: **p = 0.0013. Wilcoxon rank-sum test with Bonferroni correction: Control vs Vgat cKO: *p = 0.024; Control vs Gabra2 cKO: *p* = 0.14. **i** Schematic diagrams show the synaptic connections involved in contextual modulation in the On pathway of the direction selective circuit in wild type and Vgat cKO mice (Yellow rectangles). See also Supplementary Fig. 8 for modulation of direction-contrast in Vgat cKO mice

Compared to the weakly modulated pDSGC On responses in control (Fig. 4b) and Gabra2 cKO mice (Fig. 5b), pDSGC On responses in Vgat cKO mice showed enhanced sensitivity to phase-contrast gratings (Fig. 6a–c). Such enhancement of On contextual modulation in Vgat cKO mice was due to enhanced surround suppression during uniform grating (Fig. 6d). Consistent with pDSGC spiking response, the EPSCs of On SACs in Vgat cKO mice also showed enhanced contextual sensitivity to phase-contrast gratings (Fig. 6e–g) owing to a stronger surround suppression of EPSCs of On SACs during uniform grating (Fig. 6h). Residual inhibitory inputs onto On SACs from non-starburst amacrine cells^23^ remained insensitive to surround motion patterns (Supplementary Fig. 7). These results further support the participation of the BC – On SAC synapse in the contextual modulation of pDSGC On response: when the glutamatergic excitation of On SACs in Vgat cKO mice becomes more contextually sensitive to phase-contrast gratings (Fig. 6g), pDSGC On spiking in these mice also becomes more contextually sensitive (Fig. 6c).

No statistically significant enhancement of contextual modulation was detected between the uniform and direction-contrast gratings for either pDSGC On spiking activity or On SAC EPSCs in Vgat cKO mice (Supplementary Fig. 8). We reason that the white moving edges in the center and surround regions during the direction-contrast grating stimulus show time-dependent perpendicular distance, while the center and surround white edges of the phase-contrast grating remain maximally separated by the width of the white bar (206 μm) (see schematics in Fig. 4a). Therefore, the enhanced sensitivity to discontinuous edges in Vgat cKO mice may be more readily revealed by the phase-contrast grating than by the direction-contrast grating.

Why is contextual sensitivity in the On pathway enhanced in Vgat cKO mice? We hypothesize that GABA released from On SACs normally inhibits the contextually sensitive amacrine cell (AC)–BC–On SAC microcircuit (Fig. 6i, wild type). Disrupting GABA release from SACs in Vgat cKO mice unmasks this contextually sensitive AC–BC presynaptic inhibition, and allows for stronger contextual modulation of acetylcholine release from On SACs to pDSGCs (Fig. 6i, Vgat cKO). Functional and connectomic analysis has revealed two postsynaptic targets of On SACs: neighboring On SACs and a class of WACs^22,23^. Since On SACs do not directly inhibit BCs, the WACs postsynaptic to On SACs are the best candidate for conferring contextual sensitivity in the On pathway.

**Fig. 7:**
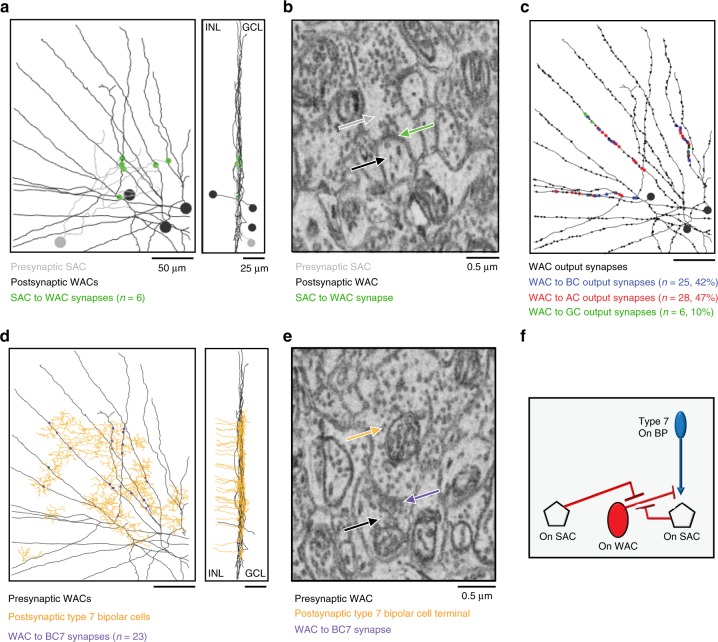
On SAC–On WAC–On BC–On SAC synaptic motif is detected by connectomic tracing. **a** Connectomic reconstruction of an On SAC dendritic sector (grey) and 3 of its postsynaptic On WACs (black). Spheres indicate the soma locations of the SAC (grey) and On WACs (black). **b** An electron microscopy (EM) example of On SAC–On WAC synapse. **c** Identity of the output synapses from the On WACs reconstructed in **a**. Color code indicates the *n* = 59 sampled synapses in which the postsynaptic partner was classified as either bipolar cell (BC), amacrine cell (AC), or ganglion cell (GC). **d** Type 7 bipolar cells previously reconstructed in the volume (orange) and the On WACs reconstructed in **a** (black). Small purple spheres indicated On WAC – BC7 synapses. **e** An electron microscopy (EM) example of On WAC–BC7 synapse. **f** Schematic summarizes the On SAC–On WAC–On BC7–On SAC synaptic connections reconstructed by connectomic tracing

To determine if the above circuit model (Fig. 6i) based on functional measurements is supported by anatomy, we performed connectomic reconstruction of the synaptic circuit postsynaptic to On SACs. We traced the amacrine cells postsynaptic to a wedge of an On SAC and focused on 3 WACs that tightly co-stratify with the ON SAC dendrites (Fig. 7a, b). Two WACs had displaced somas and one had a soma in the inner nuclear layer (INL). We identified 6 synapses between the presynaptic On SAC wedge and the 3 WACs. Notably, all 3 WACs postsynaptic to the On SAC (Fig. 7a), as well as the previously traced WACs that inhibit Off SACs (Fig. 3h)^23^, have straight, unbranched dendritic segments extending distances of hundreds of microns, making them strong candidates for detecting continuous edges (also see Discussion). Next, the output synapses of the 3 WACs were traced, and 20 synapses from each WAC were selected for further tracing of their postsynaptic partners. We found 42% of the output synapses of the WACs were made onto bipolar cell terminals (Fig. 7c). Next, we asked whether the postsynaptic bipolar cells targeted by these WACs contain bipolar cell types that are known to synapse onto On SACs. We identified 23 synapses from these three WACs to type 7 bipolar cells (Fig. 7d, e), which are well characterized as a major bipolar cell type synapsing onto On SACs^23^. Therefore, both the electrophysiological results and connectomic reconstruction support the On SAC–WAC–BC–On SAC microcircuit motif underlying the On contextual sensitivity of pDSGCs (Fig. 7f).

Similar to the Off pathway, the stronger suppression of On responses during uniform grating was eliminated or masked by new effects after applying TTX to the control retina (Supplementary Fig. 5a, c), indicating that spiking cells are involved either as the WACs or indirectly upstream of this microcircuit motif to influence center-surround interactions.

### A circuit model of pDSGC contextual modulation

**Fig. 8:**
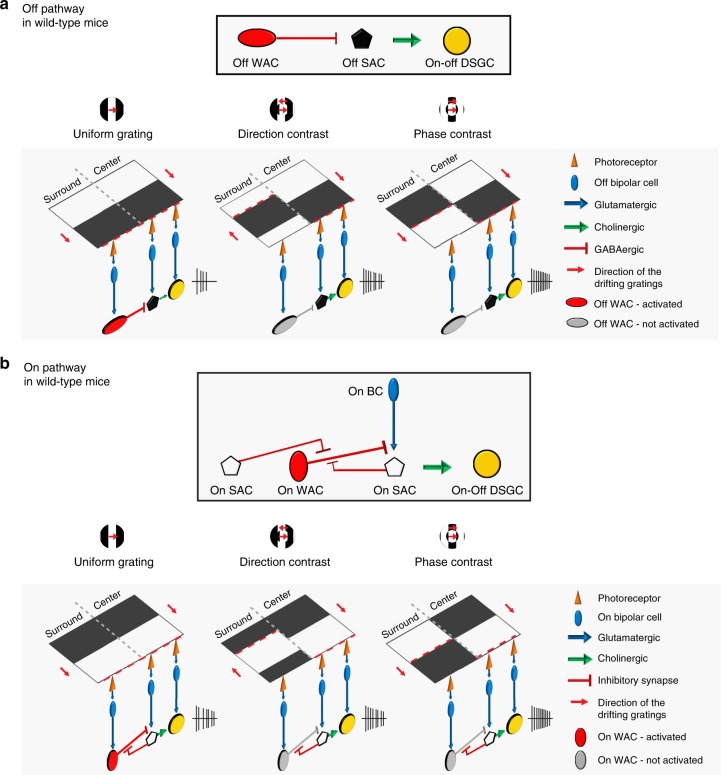
Divergent synaptic circuits implement contextual modulation in the Off and On pathways of pDSGCs. **a** A model of the synaptic mechanism underlying contextual modulation in the Off pathway. Box at the top: Schematic shows the synaptic motif in the Off pathway that participate in contextual modulation. Off WACs provide direct GABAergic inhibition onto Off SACs to suppress the cholinergic excitation from these Off SACs onto pDSGCs. Schematics in the bottom: Activation of the contextually modulated synaptic circuitry in wild type mice under different stimulus conditions. The leading edges of the black bars of the drifting gratings are labeled with red dashed lines. During uniform grating, Off WAC–Off SAC inhibition is maximally activated by the continuous dark contour and causes the strongest suppression of DSGC response. This inhibitory connection is less activated when discontinuities are present between center and surround contours during compound gratings (direction-contrast and phase-contrast). As a result, DSGC response is less suppressed during the compound gratings. Such difference between uniform grating and compound gratings is disrupted in Gabra2 KO mice in which Off WAC–Off SAC inhibition is removed. **b** A model of the synaptic mechanism underlying contextual modulation in the On pathway. Box at the top: Schematic shows the relevant synaptic connections in the On pathway. On WACs provide contextually sensitive inhibitory inputs onto bipolar cells (BCs) presynaptic to On SACs. In wild type mice, this inhibitory connection is suppressed by the GABAergic inhibition from On SACs. Schematics in the bottom: The leading edges of the white bars of the drifting gratings are labeled with red dashed lines. Because of the On SAC–On WAC–On BC–On SAC synaptic motif, the pDSGC On response is weakly modulated by stimulus contexts. Such weak contextual modulation is enhanced in Vgat cKO mice in which On SAC–On WAC inhibition is disrupted, but not affected in Gabra2 cKO mice in which GABA_A_ receptors of SACs are disrupted

Based on the above results, we propose a circuit model of pDSGC contextual modulation (Fig. 8). Since uniform grating generates stronger surround suppression of pDSGC responses than gratings with phase-contrast or direction-contrast, we hypothesize that the contextually sensitive WACs with straight, unbranched dendrites are preferentially activated by a continuous moving contour extending across the center and surround regions. Gratings with direction-contrast or phase-contrast introduce discontinuities between the center and surround regions, and therefore weaken the WAC activation.

**Fig. 9:**
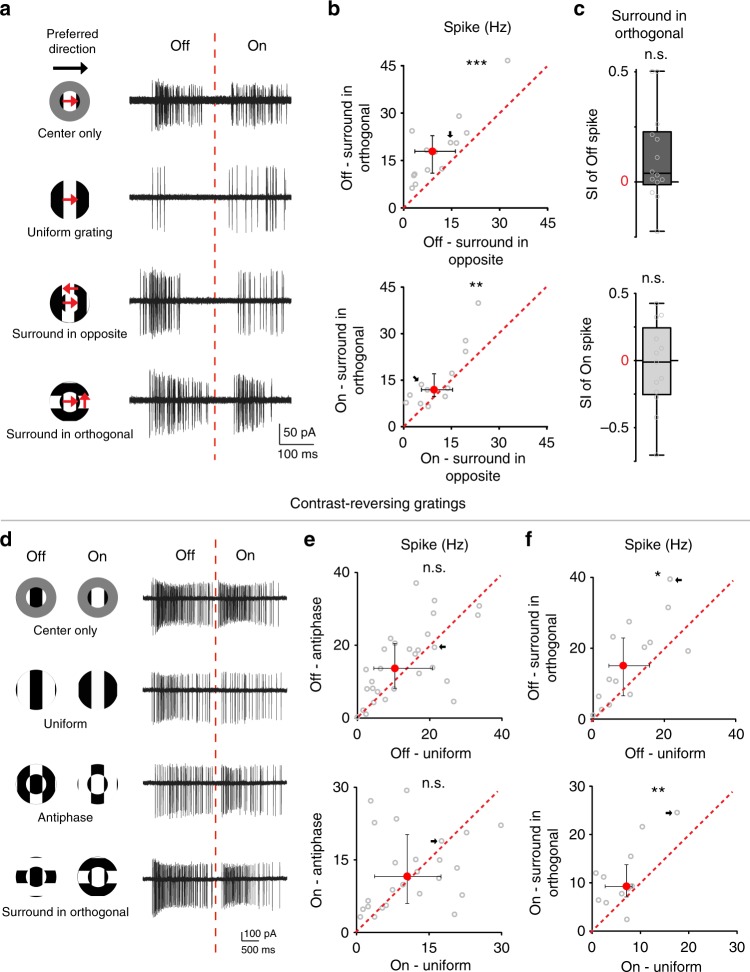
Contextual modulation of pDSGCs is sensitive to the relative orientation of center and surround contours. **a** Example spike traces of a pDSGC from a control mouse evoked by the visual stimuli shown on the left. The red dashed line shows the boundary of the Off and On responses. **b** Comparison of pDSGC firing rate during surround-in-orthogonal versus surround-in-opposite gratings, pairwise comparison was performed using Wilcoxon signed-rank test. Off response: *n* = 14 cells from 8 mice, ***p < 0.001; On response: *n* = 13 cells from 7 mice, **p = 0.0081. **c** Upper panel: SI of Off spike during surround-in-orthogonal: Wilcoxon signed-rank test was used to test whether the SI of surround-in-orthogonal is significantly higher than 0: *p* = 0.091. Lower panel: SI of On spike during surround-in-orthogonal: p = 0.32. The same sample groups as Fig. 8b. **d-f** Loose-patch recording of pDSGC under contrast-reversing stationary gratings. **d** Left: Schematics show the contrast-reversing stationary grating stimuli. Right: Spiking responses of an example pDSGC from a control mouse. Red dashed line labels the boundary of Off and On spiking. **e** Scatter plots compare pDSGC spike firing rate during uniform contrast-reversing gratings and antiphase contrast-reversing gratings. Off response: *n* = 29 cells from 7 mice, *p* = 0.14; On response: *n* = 26 cells from 7 mice, *p* = 0.70. **f** Same as **e**, but comparing pDSGC spike firing rate during uniform contrast-reversing gratings versus surround-in-orthogonal contrast-reversing gratings. Off response: *n* = 14 cells from 4 mice, *p = 0.020; On response: *n* = 11 cells from 4 mice, **p = 0.0098

Although the center and surround edges in gratings with direction-contrast and phase-contrast are discontinuous, their orientations are always the same. We next used a drifting grating stimulus in which the surround grating drifted in the orthogonal directions relative to the center grating (surround-in-orthogonal, Fig. 9a, schematics). Under this condition, the orientations of center and surround contours differ at all times. Notably, we found that this surround-in-orthogonal grating did not evoke significant surround suppression of pDSGC response relative to the center-only response (Fig. 9c). As a result, the spike response of pDSGC during the surround-in-orthogonal grating was greater than that during the direction-contrast grating (renamed as surround-in-opposite in Fig. 9a, b). Together, these results indicate that the activation of the WACs is maximal during the movement of a continuous edge spanning the center and surround, and is minimal when center and surround edges are moving in orthogonal directions.

Finally, we tested whether contextual modulation of pDSGC responses can also occur in the absence of motion. We presented three types of stationary contrast-reversing grating patterns to each pDSGC (Fig. 9d): (1) a uniform grating with center and surround gratings in the same phase (uniform), (2) a compound grating with a 180-degree center/surround phase shift (antiphase), and (3) a compound grating where the edges in the center and the surround are orthogonal to each other (surround-in-orthogonal). We found no statistically significant difference in pDSGC spiking activity between uniform and antiphase contrast-reversing gratings (Fig. 9e), in contrast to the differential responses of pDSGCs to drifting uniform and compound gratings with parallel edges (Figs 1d and 4b). We postulate that such motion sensitivity of DSGC contextual modulation likely reflects the fact that uniform drifting gratings contain continuous edges that sweep across the entire visual stimulation area, and are therefore effective in activating many WAC dendrites in the area. The continuous/discontinuous edges of the stationary uniform/antiphase gratings in our study, by contrast, are confined to fixed spatial locations, and therefore may not be as effective in differentially activating WAC dendrites as drifting gratings. However, we did found that surround-in-orthogonal contrast-reversing grating triggered a higher firing rate of the pDSGC than contrast-reversing uniform gratings (Fig. 9f). Therefore, even though the WAC-mediated circuitry plays important roles in the contextual modulation of moving stimuli, it can also contribute the center-surround interactions evoked by other non-motion visual stimuli.

## Discussion

Our study reveals that the responses of pDSGCs to bright and dark stimuli are differentially modulated by motion patterns in the surround owing to the divergent inhibitory microcircuits in the On and Off pathways. Although our study focuses on the posterior subtype of mouse On-Off DSGCs, we speculate that the mechanisms we reported here may contribute to the contextual modulation in all subtypes of On-Off DSGCs because of the following reasons. First, contextual modulation of On-Off DSGCs spiking was reported in the rabbit retina^7^, which sampled all four subtypes. No difference was reported among subtypes of On-Off DSGCs. Second, from our study, the mechanisms of pDSGC contextual modulation in both On and Off pathways act on SACs, which contribute to the spiking activity of all four types of On-Off DSGCs (reviewed by^37^).

Distinct microcircuits for contextual modulation were revealed in the On and Off pathways of the direction-selective circuit. In the Off pathway, contextual sensitivity of pDSGCs depends on the GABAergic connection from WACs to Off SACs, which in turn maximally reduces cholinergic excitation from Off SACs to pDSGCs (Fig. 8a). However, a mirror symmetric motif was not found for On SACs, and the moderate contextual modulation in the On pathway is mediated by a On SAC–WAC–BC–On SAC motif. This asymmetry may reflect the fact that On SACs also synapse onto On DSGCs^38,39^, which signal global image shift rather than moving objects against their backgrounds^40,41^. The contextual sensitivity of On DSGCs to motion coherence has not been determined, and remains an interesting topic for future investigation.

A new function of On SACs was demonstrated here: GABA release from On SACs inhibits a population of WACs that provide contextual sensitivity to On SACs (Fig. 8b). Functional and connectomic data both support a previously unidentified On SAC–WAC–BC–On SAC circuit motif underlying this contextual modulation. In contrast, Off SACs do not make considerable synapses onto WACs^23^. These results together highlight the power of combining functional and connectomic methods in neural circuit analysis.

Despite distinct postsynaptic targets of WACs in the On and Off pathways for pDSGC contextual modulation (Fig. 8), the WACs synaptically connected to On and Off SACs show pronounced similarities in their dendritic morphology: they all have straight, unbranched dendritic segments extending over long distances. This morphological feature, in contrast to a more branched and space-filling dendritic arbor such as those of pDSGCs, is uniquely suitable for detecting continuities of contours in visual stimuli. A continuous moving edge over the retina likely activates the BCs presynaptic to these WAC dendritic branches simultaneously, resulting in strong dendritic activation. In contrast, these WAC dendrites receive less synchronized excitation when the contours contain discontinuities, and thereby reduce the inhibitory control of their postsynaptic targets. An analogous mechanism has been implicated in the salamander retina, in which the long, straight axons of polyaxonal amacrine cells exert stronger inhibition during global motion than during differential motion^33^. It is noteworthy that although the WAC circuitry underlies motion-evoked contextual sensitivity of pDSGCs, motion is not exclusively required for the differential activation of these WACs, since contextual modulation of pDSGCs is also present during stationary contrast-reversing gratings (Fig. 9f). Future development of novel tools and methods for directly recording these WACs will provide deeper insights into their RF properties.

Connectomic analysis shows that On WACs provide inputs to multiple cell types including BCs, ACs and ganglion cells. Therefore, it is possible that these “continuity detectors” participate in visual processing of multiple retinal circuits besides the direction-selective circuit. Furthermore, since On SACs inhibit the WACs in the On pathway, our study raises an interesting possibility that On SACs may be involved in the contextual sensitivity of multiple retinal ganglion cell types in addition to its renowned role in generating direction selectivity in DSGCs. We noted that a small fraction of presynaptic inputs from On WACs to On SACs (5%) was also reported by a previous study^23^. However, contextual sensitivity of pDSGC On responses is not affected in Gabra2 cKO mice, suggesting that these On WACs-On SAC connections alone are not sufficient to modulate contextual sensitivity of pDSGCs. It is not clear how these WACs contribute to pDSGC responses, and if these WACs are also inhibited by On SACs. Future connectomic analysis will provide deeper insights into this question.

Are direction selectivity and contextual sensitivity of On-Off DSGCs independent of each other? Our study shows that this question needs to be addressed separately for On and Off pathways. The Off response of pDSGCs shows pronounced contextual sensitivity at the expense of robust direction selectivity, because the preferred-, but not null-direction Off response is more drastically suppressed by uniform grating than by compound gratings. On the other hand, the On response of pDSGCs maintains robust direction selectivity at the expense of contextual sensitivity, because the preferred-direction On response is moderately and similarly suppressed by compound gratings and uniform grating, leading to similar DSI values of pDSGC On responses across these stimulus conditions.

We postulate that these divergent RF properties of pDSGC On and Off responses may reflect the adaptation to the light/dark asymmetries in the natural environment^42^. Enhanced sensitivity to image discontinuities in the Off pathway and robust direction selectivity in the On pathway may also confer an advantage in information encoding of On-Off DSGCs by reducing the redundancy of information in the On and Off channels. Indeed, asymmetries between the On and Off pathways of the retina have been implicated in efficient motion estimation of natural scenes in both humans and flies^43,44^.

On-Off DSGCs in the mouse retina innervate the superior colliculus (SC) and the dorsal lateral geniculate nucleus (dLGN)^16,45–47^, two major nuclei involved in the image-forming pathway and visually guided behavior. In the superior colliculus, the direction selectivity of collicular neurons originates from direction-selective inputs from DSGCs, and selective ablation of retinal direction selectivity leads to a loss of collicular direction selectivity^48^. However, although collicular neurons inherit their direction selectivity from On-Off DSGC inputs, they do not inherit exactly the same contextual modulation pattern of On-Off DSGCs^49^. Differential contextual effects of surround motion in retinal and collicular direction-selective neurons provide a remarkable example of signal transformation along the visual pathways, and a starting point for investigating the input-output relationships for complex motion stimuli in higher visual circuits.

Finally, we noted that the contextual modulation of On-Off DSGCs exhibits interesting mechanistic similarities to that of visual cortical neurons. It has been well-documented in V1 neurons that surround suppression is selectively engaged by statistical redundancy between center and surround images^50–52^. The suppression of spiking activity for visual cortical neurons is accompanied by reduced excitatory drive and a concomitant reduction of inhibition^53,54^, similar to what was observed in On-Off DSGCs in the retina (Fig. 2). It is yet to be explored if a unified model can account for contextual modulation in multiple visual areas despite the differences in their detailed cellular and synaptic organizations.

## Methods

### Mice

The *Gabra2*^*flox/flox*^ mouse line was a generous gift from Dr. Uwe Rudolph at Harvard Medical School. *Vgat*^*flox/flox*^ mice (*Slc32a1* *<* *tm1Lowl* *>* */J*), *Chat-IRES-Cre* mice (129S6-Chat^tm2(cre)Lowl^/J) and floxed *tdTomato* mice (129S6*-Gt(ROSA)26Sor*^*tm9(CAG-tdTomato)Hze*^/J) were acquired from the Jackson Laboratory. *Drd4–GFP* mice were originally developed by MMRRC (http://www.mmrrc.org/strains/231/0231.html) in the Swiss Webster background, and were subsequently backcrossed to C57BL/6 background. All strains were backcrossed to the *C57BL/6* background in our laboratory, and crossed to each other to create the lines used in this study. Mice of ages P22-P68 of either sex were used. All procedures to maintain and use mice were in accordance with the University of Chicago Institutional Animal Care and Use Committee (Protocol number ACUP 72247) and in conformance with the NIH Guide for the Care and Use of Laboratory Animals and the Public Health Service Policy.

Control mice with normal retinal circuitry contain the following transgenes: Drd4-GFP for labeling of On-Off DSGCs, and ChAT-Cre and floxed tdTomato for labeling of SACs. *Vgat* conditional KO mice contain additional homozygous *Vgat*^*flox/flox*^ alleles. *Gabra2* conditional KO mice contain additional homozygous *Gabra2*^*flox/flox*^ alleles.

### Whole-mount retina preparation

After dark adaptation for > 30 min, mice were anesthetized with isoflurane and euthanized by decapitation. Retinas were isolated from the pigment epithelium under infrared illumination at room temperature in oxygenated Ames’ medium (Sigma-Aldrich, St. Louis, MO). The retinas were then cut into halves and mounted ganglion-cell-layer-up on top of a ~ 1.5 mm^2^ hole in a small piece of filter paper (Millipore, Billerica, MA). Cells in the center of the hole were used for experiments. The mounted retinas were kept in darkness at room temperature in Ames’ medium bubbled with 95% O_2_/5% CO_2_ until use (0–8 h).

### Visual stimulation

A white organic light-emitting display (OLEDXL, eMagin, Bellevue, WA; 800 × 600 pixel resolution, 60 Hz refresh rate) was controlled by an Intel Core Duo computer with a Windows 7 operating system and was presented to the retina at a resolution of 1.1 μm/pixel. All stimuli were generated by MATLAB and the Psychophysics Toolbox^55^, projected through the condenser lens of the two-photon microscope focusing on the photoreceptor layer, and centered to the cell somas.

The diameter of center stimuli is 330 μm to ensure complete coverage of the receptive field center for all pDSGCs with diverse dendritic morphology^16,20^. This center size is also near the peak of spatial tuning curve of mouse On-Off DSGCs using bright spots of increasing size^32,56,57^. The surround diameter is 660 um, which is the maximum diameter in our visual stimulus setup and evokes significant suppression of the center response^32,56^. The spatial (0.08 cycle/degree) and temporal frequencies (2 cycles/second) of the gratings lie in the optimal range for mouse On-Off DSGCs to maximize the center response in the preferred direction^32^. These frequencies correspond to alternating bright and dark bars spaced at 206 μm moving at a speed of 824 μm/s over the retina. Contrast-reversing gratings share the same size and spatial frequency as drifting gratings, and the duration of bright and dark bars is 1 s respectively.

All experiments used a mean light intensity of ~ 1.6 × 10^5^ isomerizations (R*)/rod/s in the photopic range. 100% square wave grating with a periodicity of 412 μm was used for both drifting grating and contrast-reversing grating stimuli. The light intensity for the white bar of the grating was ~ 3.2 × 10^5^ isomerizations (R*)/rod/s. The drifting grating moves at 2 Hz with duration of 3 s (6 grating cycles in total). In each set of stimuli, center gratings were always moving in the same direction while different stimulus contexts were presented in 3–6 trials of pseudorandomized sequence by Matlab. When the direction of center drifting grating was altered in preferred or null directions, these two sets of stimuli were presented in random sequence as well. The inter-trial-interval is 2 s. The light intensity of the gray area used in center-only and surround-only stimuli is the mean luminance of the square wave grating. A gray background with the same light intensity was used before stimuli for 10 s adaptation and also at inter-stimulus interval.

### Cell targeting for electrophysiology

Cells were visualized with infrared light and an IR-sensitive video camera (Watec). GFP-labelled pDSGCs in Drd4-GFP mice and floxed-tdTomato-labelled SACs in *Chat-IRES-Cre* mice were targeted using a two-photon microscopy (Scientifica) and a Ti:sapphire laser (Spectra-Physics) tuned to 920 nm. DSGC identity was further confirmed physiologically by electrophysiological recordings of the responses to drifting grating and/or anatomically using internal solution containing 25 μM Alexa Fluo 594 (Life Technologies) to show the bistratified dendritic arbor and the cofasciculation with tdTomato-expressing SACs. For wild type mouse, the preferred direction of pDSGCs was determined physiologically. We also noted the anatomical direction during the dissection, and physiologically measured preferred direction matched well with anatomical nasal direction, as reported by many other studies on this mouse line such as^16,20,47,58^. For Vgat cKO and Gabra2 cKO mouse where direction selectivity is impaired^20,24^, the preferred direction of pDSGC was determined anatomically.

### Electrophysiology recordings

Data were acquired using PCLAMP 10 recording software and a MultiClamp 700B amplifier (Molecular Devices, Sunnyvale, CA), low-pass filtered at 4 kHz and digitized at 10 kHz. Retina was kept in Ames’ medium with a bath temperature of 32–34 °C. Recording electrodes of 3.5–5 MΩ were filled with Ames’ medium for loose cell-attached recordings or a cesium-based internal solution for whole-cell recordings, which contains 110 mM CsMeSO4, 2.8 mM NaCl, 4 mM EGTA, 5 mM TEA-Cl, 4 mM adenosine 5′-triphosphate (magnesium salt), 0.3 mM guanosine 5′-triphosphate (trisodium salt), 20 mM HEPES, 10 mM phosphocreatine (disodium salt), 5 mM N-Ethyllidocaine chloride (QX314) (Sigma), pH 7.25. Light-evoked EPSCs and IPSCs in pDSGCs and SACs were isolated by holding the cells at reversal potentials (0 mV for GABAergic and −60 mV for cholinergic). Liquid junction potential (~ 10 mV) was corrected. To investigate the contribution of different synaptic inputs, synaptic blockers 0.008 mM Dihydro-b-erythroidine hydrobromide (DHβE; from Tocris, Cat#2349; CAS: 29734-68-7) or 0.001 mM tetrodotoxin (TTX; from Tocris, Cat#1078; CAS: 4368-28-9) were used to selectively block the nicotinic acetylcholine receptors or voltage-gated Na^+^ channels respectively.

### Analysis of electrophysiological data

Data were averaged across 3–6 trials to obtain the mean response for each stimulus condition. Responses during the second to the sixth grating cycles were averaged and the responses during the first cycle were discarded to avoid the impact of wake-up responses to the onset of the stimulus. On and Off responses were separated based on the time window of the visual stimulus. For all the drifting grating stimuli, the center gratings always start at the same spatial location relative to the pDSGC, with the left dark edge moving from outside the outer rim of the pDSGC dendritic field (103 um away from the soma) toward the center. During the five grating cycles in each trial (500 ms per cycle), we used alternating 250 ms windows for Off and On responses based on the periodic Off and On spiking and PSP waveforms. The same time window was used for both spiking and PSC recordings, and for all drifting grating stimuli in all mouse lines. The start of the Off window in each cycle corresponds to the center dark edge moving ~ 45 μm into the pDSGC dendritic field. For both spike and PSC recordings, responses were first averaged across trials, and then responses during second to sixth grating cycles were averaged.

For loose cell-attached recordings, data were analyzed using custom protocols in MATLAB to count the total number of spikes within each 250 ms On or Off window. Small spike-counts of center-only response is difficult to quantify further suppressive contextual modulation effects since the baseline response is too low to begin with. Therefore, for On or Off spiking datasets, cells with < 10 spike/trial during center-only stimulus were discarded. For whole-cell voltage clamp recordings, data were analyzed using PCLAMP 10 software to obtain the peak amplitude and total charge transfer of EPSCs and IPSCs. Membrane tests were performed to check the whole-cell recording quality, and recordings with series resistances > 25 MΩ were discarded. Correlation between EPSC waveform and the spiking activity was calculated based on PSTH with 100-ms bin size.

Suppression index (SI) was used to quantify the suppression strength, which is defined as:${\mathrm{SI}} = \frac{{N_{{\mathrm{center}} - {\mathrm{only}}\;{\mathrm{grating}}} - N_{{\mathrm{full}} - {\mathrm{field}}\;{\mathrm{grating}}}}}{{N_{{\mathrm{full}} - {\mathrm{field}}\;{\mathrm{grating}}} + N_{{\mathrm{center}} - {\mathrm{only}}\;{\mathrm{grating}}}}}$, where *N* is the firing rate. A higher positive SI value indicates stronger suppression, while a negative SI value indicates facilitation. Direction selective index (DSI) was used to quantify the direction selectivity, which is defined as ${\mathrm{DSI}} = \frac{{N_{{\mathrm{preferred}}\;{\mathrm{direction}}} - N_{{\mathrm{null}}\;{\mathrm{direction}}}}}{{N_{{\mathrm{preferred}}\;{\mathrm{direction}}} + N_{{\mathrm{null}}\;{\mathrm{direction}}}}}$, where *N* is the firing rate. Contextual modulation index (CMI) was used to quantify the level of contextual modulation, which is defined as:${\mathrm{CMI}} = \frac{{N_{{\mathrm{compound}}\;{\mathrm{grating}}} - N_{{\mathrm{uniform}}\;{\mathrm{grating}}}}}{{N_{{\mathrm{compound}}\;{\mathrm{grating}}} + N_{{\mathrm{uniform}}\;{\mathrm{grating}}}}}$, where *N* is the firing rate. Compound grating here refers to direction-contrast or phase-contrast. For On SAC results, SI and CMI were calculated in the same way but with charge transfer instead of firing rate.

### Connectomic reconstruction/Skeleton tracing and contact annotation

A previously published dataset acquired using scanning SBEM was analyzed (retina k0725; Ding et al.^23^). Voxel dimensions were 13.2 × 13.2 × 26 nanometer (nm) (*x*, *y*, and *z*, respectively). On sublamina WACs were first identified by exploratory tracing of dendrites postsynaptic to an On SAC. The dendrites of On WACs were readily distinguishable from postsynaptic On SACs due to the relatively straight trajectories of their dendrites. The presynaptic release sites along the dendrites of 3 On WACs (whose somata were also found within the EM volume) were then manually annotated. We sampled 59 of these release sites and classified the postsynaptic partners classified as either ganglion cell (*n* = 6, 10%), amacrine cell (*n* = 28, 47%) or bipolar cell (*n* = 25, 42%).

We then examined proximity locations between the On WAC dendrites and previously reconstructed type 7 bipolar cells (Ding et al.^23^) and identified 23 synapses between the On WACs and type 7 bipolar cells. Because we did not perform a complete sampling and only targeted the analysis to type 7 bipolar cell terminals, we cannot rule out whether the On WAC dendrites additionally synapse onto additional bipolar cell terminals.

All analyses were performed by tracing skeletons and annotating synapses using the Knossos software package (https://knossostool.org/, Helmstaedter et al.^59^).

### Statistical analysis

Statistical comparisons were performed using Wilcoxon signed-rank test for single samples and paired samples or Wilcoxon rank-sum test for group samples. For multiple comparisons, Friedman test was used for repeated measurements while Kruskal–Wallis test was used for non-repeated measurements, and both were followed by post-hoc tests with Bonferroni correction. *p* < 0.05 was considered significant. *p < 0.05; **p < 0.01; ***p < 0.001.

### Reporting summary

Further information on research design is available in the Nature Research Reporting Summary linked to this article.

## Supplementary information

Supplementary Information

Reporting Summary

Description of Additional Supplementary Information

Supplementary Movie 1

## Data Availability

All relevant data generated and analyzed in this study are available from the authors on reasonable request.
