# Supplementary material for: Neural mechanisms of contextual modulation in the retinal direction selective circuit
Source: Nat Commun. 2019 Jun 3;10:2431. doi: 10.1038/s41467-019-10268-z (PMC6547848; doi:10.1038/s41467-019-10268-z)
Supplement: Supplementary file 1 — Supplementary Information [file 41467_2019_10268_MOESM1_ESM.pdf]

Supplementary Information

Neural mechanisms of contextual modulation in the retinal direction selective circuit

Huang et al

Supplementary Figure 1

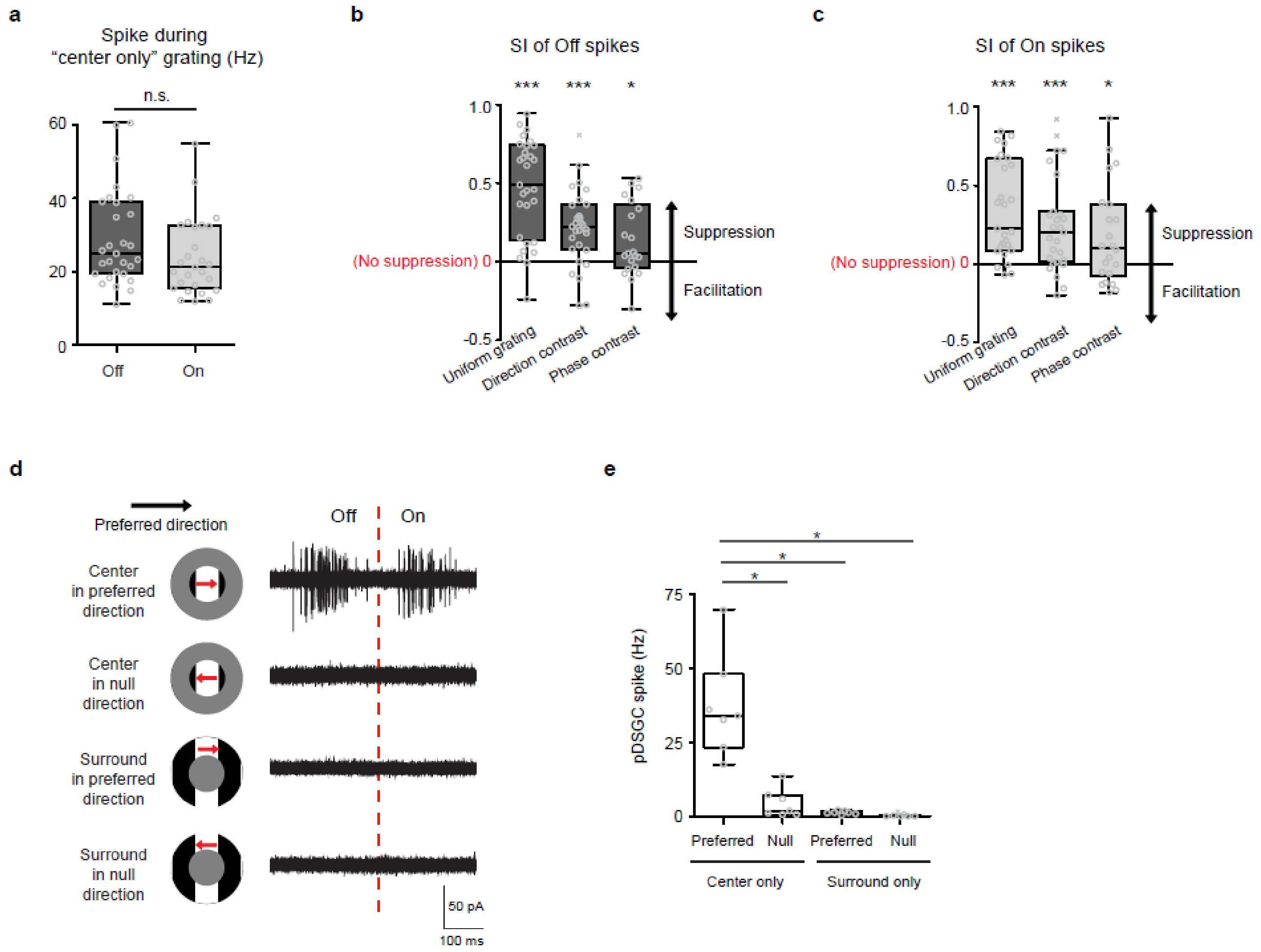

Supplementary Figure 1. pDSGCs have suppressive RF surrounds.

a, Comparison of Off and On spike firing rate during “center only” stimulus. Wilcoxon signed-rank test, p = 0.079, the same sample groups as Figures 1d and 4b.

b and c, Suppression index (SI) of pDSGC Off and On spikes in different motion contexts. Suppression index (SI) of DSGC spiking was calculated as ($N_{\text{center only grating}}$ - $N_{\text{full-field grating}}$) / ($N_{\text{center only grating}}$ + $N_{\text{full-field grating}}$), where N is the spike firing rate. An SI of 0 indicates no suppression, an SI > 0 indicates surround suppression, and an SI < 0 indicates surround facilitation. Wilcoxon signed-rank test was used to test whether the SI value is significantly higher than 0.

b, Off SI, the same sample group as Figure 1d. Wilcoxon signed-rank test was used to test whether the SI of “surround in orthogonal” is significantly higher than 0: uniform grating: ***p < 0.001; direction-contrast: ***p < 0.001; phase-contrast: *p = 0.033.

c, On SI, the same sample group as Figure 4b: uniform grating: ***p < 0.001; direction-contrast: ***p < 0.001; phase-contrast: *p = 0.050.

d and e, Surround grating alone does not evoke pDSGC spiking regardless of motion directions.

d, Left: Schematic diagrams show the "center only" and the "surround only" stimuli. Right: Example spike traces of a pDSGC from a control mouse. Red dashed line shows the boundary of the Off and On spiking.

e, Summary of pDSGC spike firing rate under the visual stimuli conditions shown in **c**. n = 7 cells. "Center only" in preferred direction vs the other three stimuli, *p = 0.016.

Supplementary Figure 2

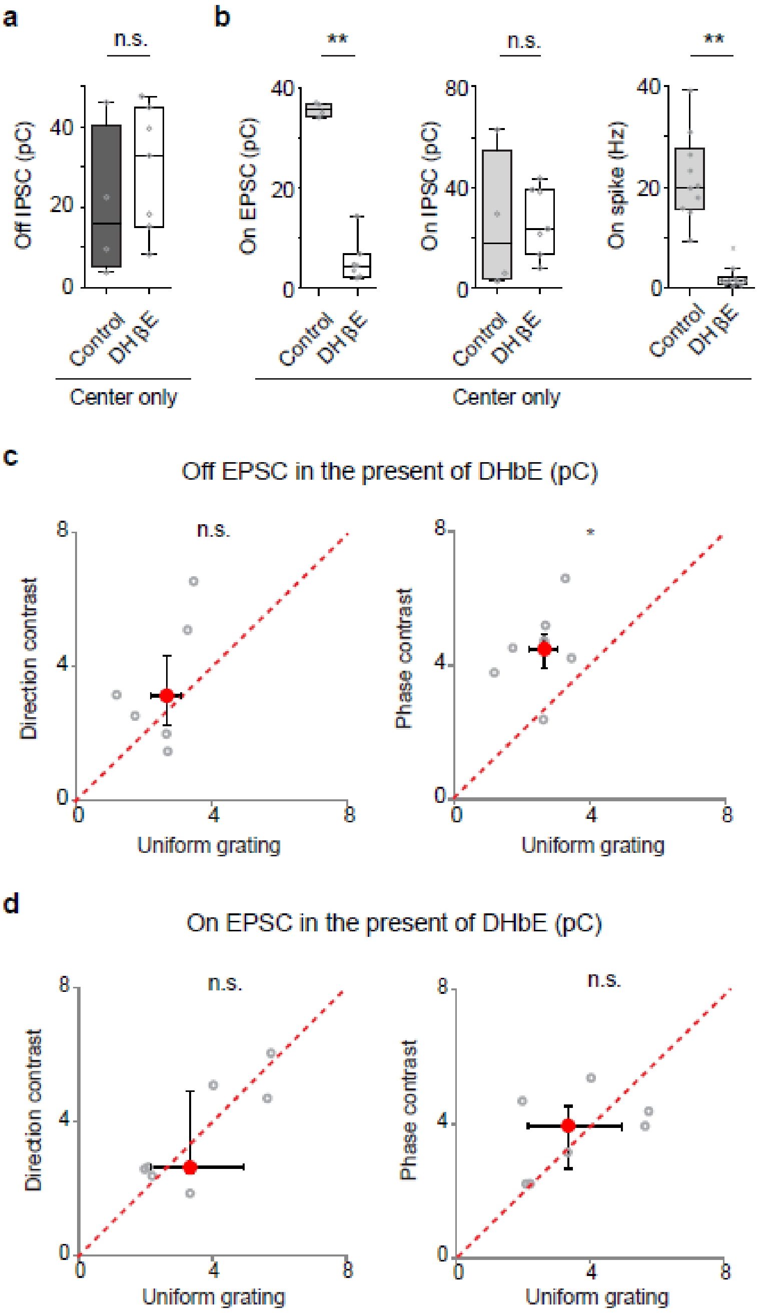

Supplementary Figure 2. Contextual modulation was not detected in the weak pDSGC responses in the presence of DHβE.

a, Summary graph of pDSGC Off inhibitory charge transfer in control (Ames' solution) or in the presence of DHβE: Wilcoxon rank-sum test, $p = 0.53$, the same sample group as pDSGC Off excitatory charge transfer in Figure 2d.

b, Left: Summary graph of pDSGC On excitatory charge transfer in control (Ames' solution, $n = 4$ cells from 2 mice) or in the presence of DHβE ($n = 7$ cells from 2 mice): Wilcoxon rank-sum test, ** $p = 0.0061$. Middle:

Summary graph of pDSGC On inhibitory charge transfer in control (Ames' solution) or in the presence of DHβE: Wilcoxon rank-sum test, p = 0.65; the same sample group as pDSGC On excitatory charge transfer on the left. Right: Summary graph of pDSGC On spike firing rate before (in Ames' solution) or after adding DHβE: n = 10 cells from 6 mice, Wilcoxon signed-rank test, ** p = 0.0020.

c, Scatter plots compare Off EPSC charge transfer between uniform grating and compound gratings in the presence of DHβE, n = 7 cells from 2 mice, the same sample group as Figure 2d. Left: Direction-contrast vs uniform grating: Wilcoxon signed-rank test, p = 0.2188. Right: Phase-contrast vs uniform grating: *p = 0.031.

d, Scatter plots compare On EPSC charge transfer between uniform grating and compound gratings in the presence of DHβE, n = 7 cells from 2 mice, the same sample group as Supplementary Figure 2b. Left: Direction-contrast vs uniform grating: Wilcoxon signed-rank test, p = 0.8125. Right: Phase-contrast vs uniform grating: p = 0.94.

Supplementary Figure 3

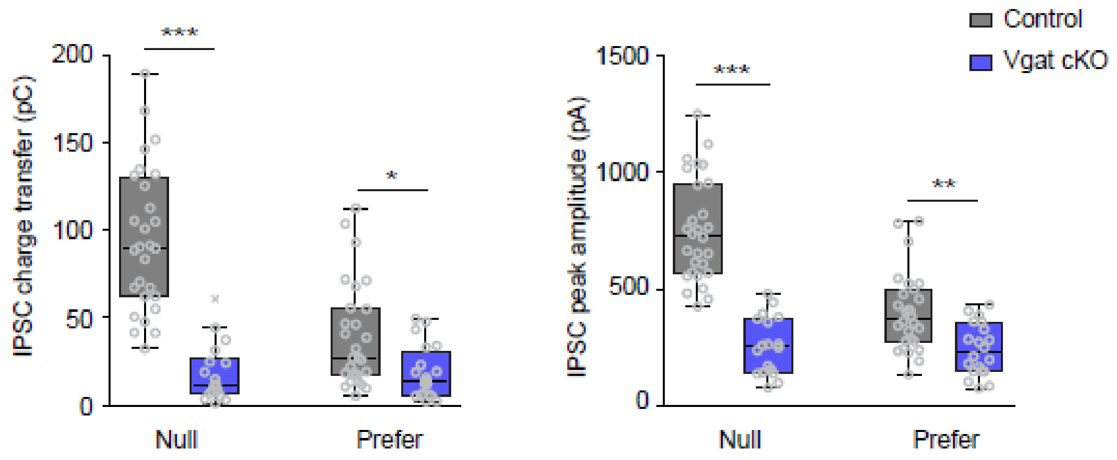

Supplementary Figure 3. pDSGC IPSCs are diminished and non-directional in Vgat cKO mice.

Left: Summary of pDSGC IPSC charge transfer during center only gratings in control and Vgat cKO mice. Center gratings moving in null direction: control: n = 28 cells; Vgat cKO: n = 18 cells; Wilcoxon rank-sum test, ***p < 0.001. Center gratings moving in preferred direction: control: n = 28 cells; Vgat cKO: n = 20 cells; Wilcoxon rank-sum test, *p = 0.0077.

Right: Summary of pDSGC IPSC peak amplitude during center only gratings in control and Vgat cKO mice. Center gratings moving in null direction: control: n = 28 cells; Vgat cKO: n = 18 cells; Wilcoxon rank-sum test, ***p < 0.001. Center gratings moving in preferred direction: control: n = 28 cells; Vgat cKO: n = 20 cells; Wilcoxon rank-sum test, **p = 0.0013.

Supplementary Figure 4

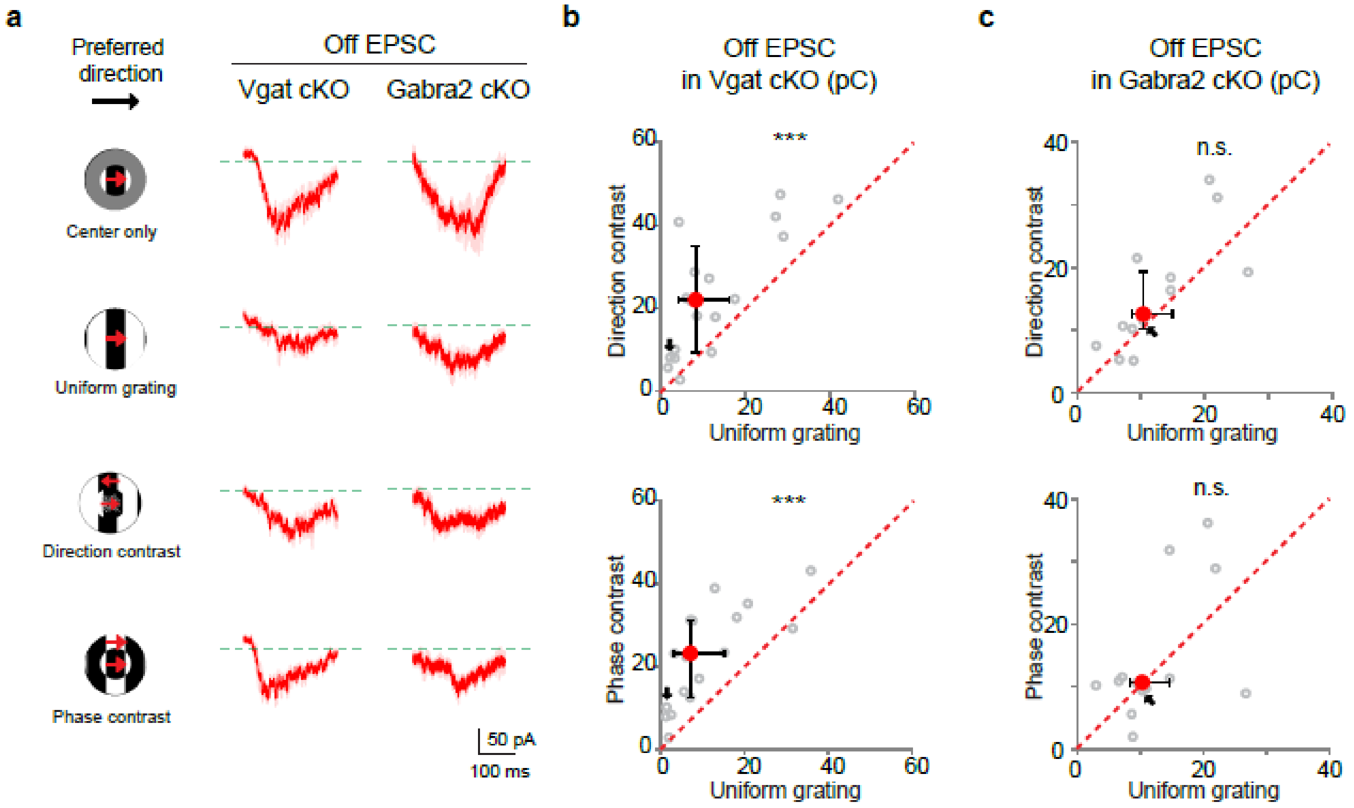

Supplementary Figure 4. Context-sensitive Off EPSCs of pDSGCs are unaffected in Vgat cKO mice, but disrupted in Gabra2 KO mice.

a, Example EPSC traces of a pDSGC from a Vgat cKO mouse or a Gabra2 cKO mice during different grating stimuli.

b, Scatter plots of pDSGC Off EPSC charge transfer in Vgat cKO mice. Upper panel: Direction-contrast vs uniform grating: n = 18 cells from 13 mice, Wilcoxon signed-rank test, ***$p < 0.001$. Lower panel: Phase-contrast vs uniform grating: n = 17 cells from 11 mice, Wilcoxon signed-rank test, ***$p < 0.001$.

c, Same as **b**, but for pDSGC Off EPSC charge transfer in Gabra2 cKO mice. Upper panel: Direction-contrast vs uniform grating: n = 13 cells from 9 mice, Wilcoxon signed-rank test, p = 0.0681. Lower panel: Phase-contrast vs uniform grating: n = 13 cells from 9 mice, Wilcoxon signed-rank test, p = 0.45.

Supplementary Figure 5

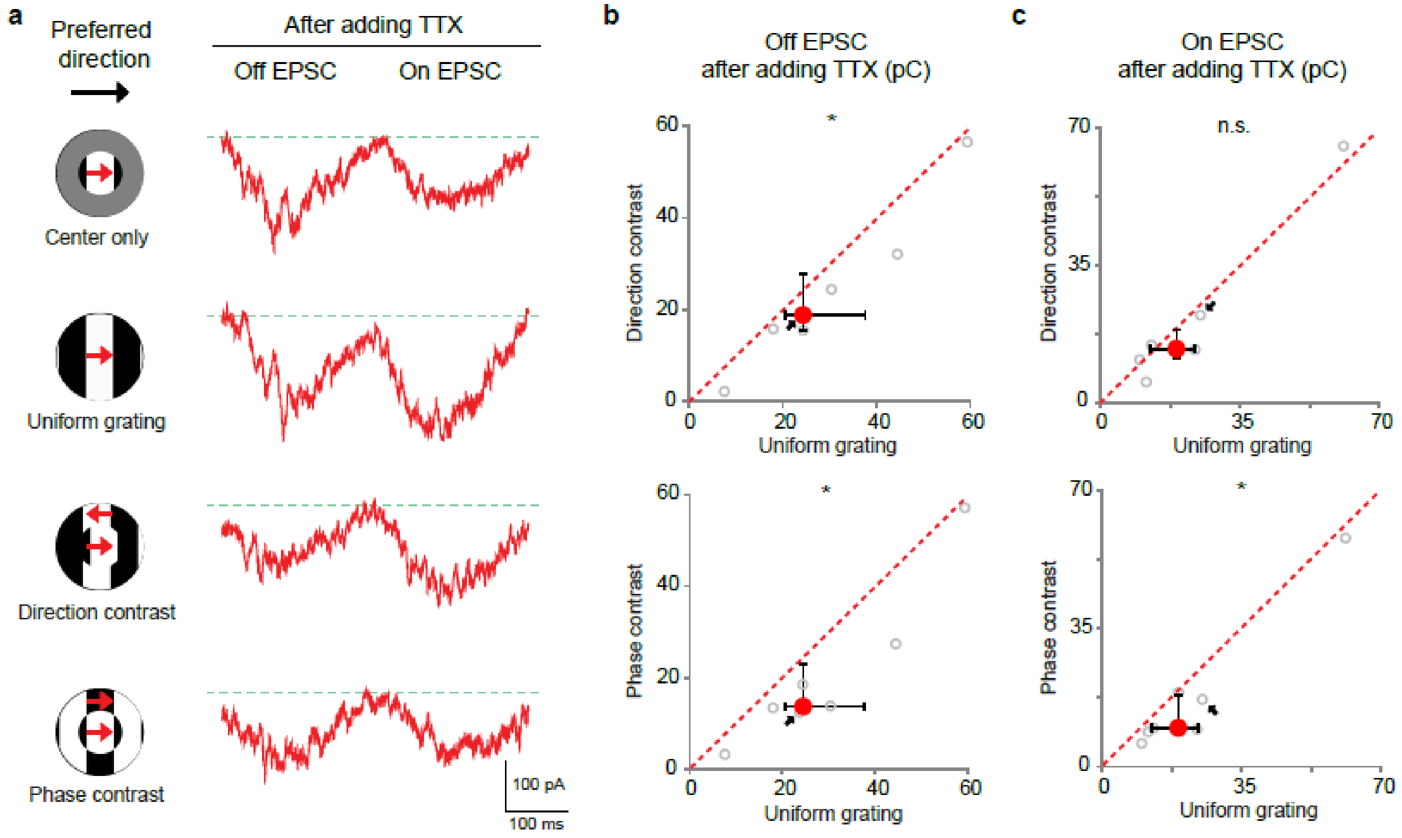

Supplementary Figure 5. Stronger suppression of pDSGC EPSCs during uniform grating is disrupted or masked in control mice after adding TTX.

a, Example traces of pDSGC EPSCs in bath of TTX.

b, Scatter plots of pDSGC Off EPSC charge transfer after applying TTX, n = 7 cells from 2 mice. Upper panel: Direction-contrast vs uniform grating: Wilcoxon signed-rank test, *p = 0.016. Lower panel: Phase-contrast vs uniform grating: Wilcoxon signed-rank test, *p = 0.016.

c, Same as **b**, but for pDSGC On EPSCs. Upper panel: Direction-contrast vs uniform grating: Wilcoxon signed-rank test, p = 0.30. Lower panel: Phase-contrast vs uniform grating: Wilcoxon signed-rank test, *p = 0.016.

Supplementary Figure 6

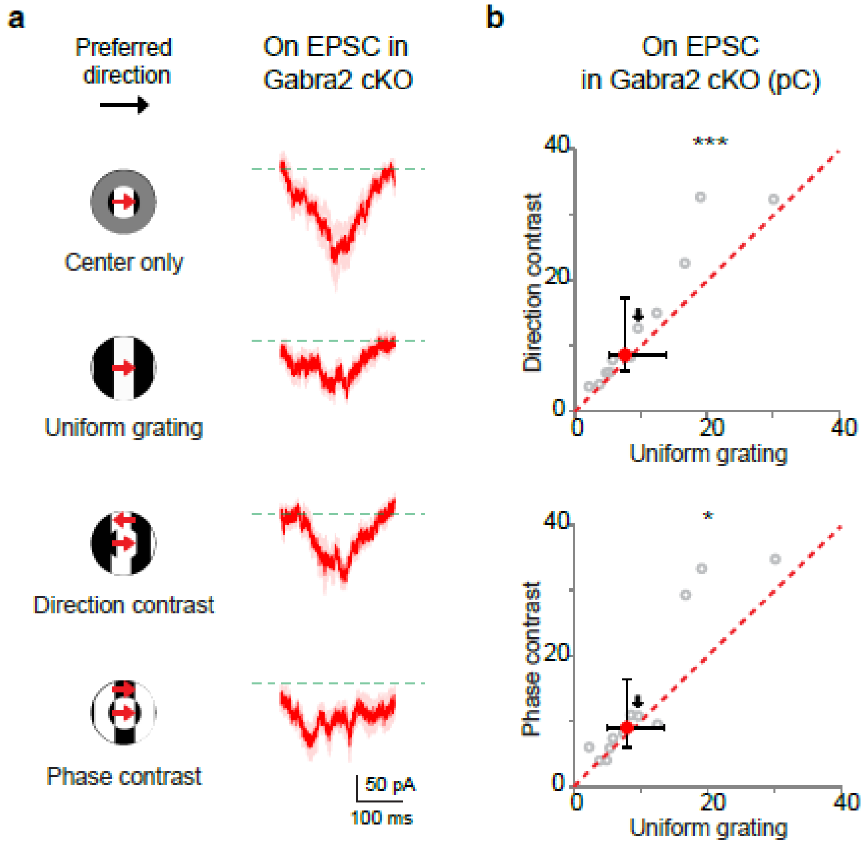

Supplementary Figure 6. Contextual modulation of pDSGC On EPSCs is unchanged in Gabra2 cKO mice.

a, EPSC traces of a pDSGC from a Gabra2 cKO mouse during different motion contexts.

b, Scatter plots of pDSGC On EPSC charge transfer in Gabra2 cKO mice. Upper panel: Direction-contrast vs uniform grating: n = 12 cells from 8 mice, Wilcoxon signed-rank test, ***p < 0.001. Lower panel: Phase-contrast vs uniform grating: n = 12 cells from 8 mice, Wilcoxon signed-rank test, *p = 0.034.

Supplementary Figure 7

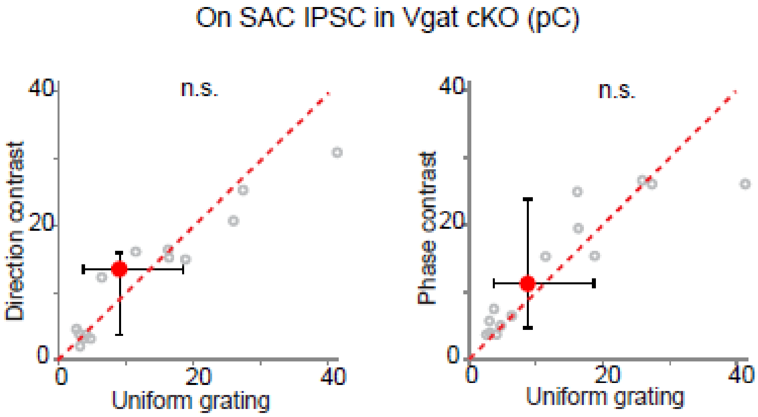

Supplementary Figure 7. In Vgat cKO mice, IPSC of On SAC during uniform grating is similar to that during compound gratings.

Scatter plots of On SAC inhibitory charge transfer in Vgat cKO mice, n = 14 cells from 5 mice. Left: Direction-contrast vs uniform grating: Wilcoxon signed-rank test, p = 0.36. Right: Phase-contrast vs uniform grating: Wilcoxon signed-rank test, p = 0.27.

Supplementary Figure 8

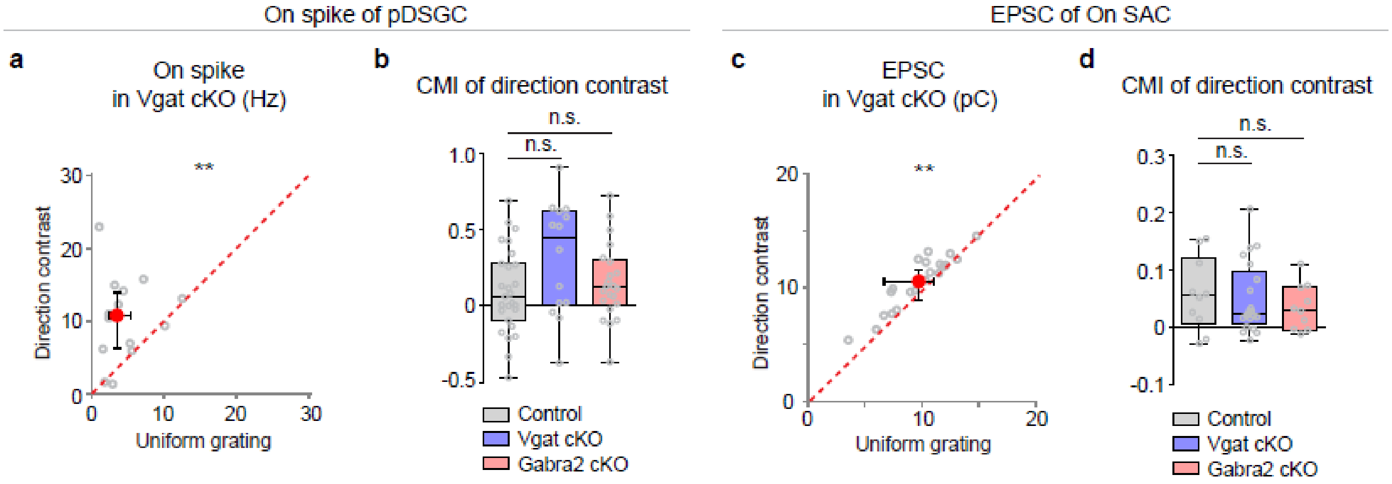

Supplementary Figure 8. Differential responses during uniform grating and direction-contrast gratings are not significantly enhanced in Vgat cKO mice.

a, Scatter plot compares pDSGC On spike firing rate during uniform grating and direction-contrast in Vgat cKO mice: n = 14 cells from 8 mice, Wilcoxon signed-rank test, **p = 0.0065.

b, Summary graph compares CMI of direction-contrast for pDSGC On response in control, Vgat cKO and Gabra2 cKO mice: the same sample group as Figure 4b (control), Supplementary Figure 8a (Vgat cKO) and Figure 5b (Gabra2 cKO), Kruskal-Wallis test, p = 0.14.

c, Scatter plot of On SAC EPSC charge transfer during uniform grating versus direction-contrast grating in Vgat cKO mice: n = 21 cells from 5 mice, Wilcoxon signed-rank test, **p = 0.0015.

d, Summary graph compares CMI of direction-contrast for EPSC charge transfer of On SAC in control, Vgat cKO and Gabra2 cKO mice: the same sample group as Figure 5d (control), Supplementary Figure 8c (Vgat cKO) and Figure 5e (Gabra2 cKO), Kruskal-Wallis test, p = 0.80.
