# Supplementary material for: Neural mechanisms of contextual modulation in the retinal direction selective circuit
Source: Nat Commun. 2019 Jun 3;10:2431. doi: 10.1038/s41467-019-10268-z (PMC6547848; doi:10.1038/s41467-019-10268-z)
Supplement: Supplementary file 2 — Reporting Summary [file 41467_2019_10268_MOESM2_ESM.pdf]

nature research

Corresponding author(s): Wei Wei

Reporting Summary

Nature Research wishes to improve the reproducibility of the work that we publish. This form provides structure for consistency and transparency in reporting. For further information on Nature Research policies, see Authors & Referees and the Editorial Policy Checklist.

Statistical parameters

When statistical analyses are reported, confirm that the following items are present in the relevant location (e.g. figure legend, table legend, main text, or Methods section).

n/a	Confirmed	
☐	☒	The exact sample size (*n*) for each experimental group/condition, given as a discrete number and unit of measurement
☐	☒	An indication of whether measurements were taken from distinct samples or whether the same sample was measured repeatedly
☐	☒	The statistical test(s) used AND whether they are one- or two-sided<br>*Only common tests should be described solely by name; describe more complex techniques in the Methods section.*
☒	☐	A description of all covariates tested
☐	☒	A description of any assumptions or corrections, such as tests of normality and adjustment for multiple comparisons
☐	☒	A full description of the statistics including central tendency (e.g. means) or other basic estimates (e.g. regression coefficient) AND variation (e.g. standard deviation) or associated estimates of uncertainty (e.g. confidence intervals)
☐	☒	For null hypothesis testing, the test statistic (e.g. *F*, *t*, *r*) with confidence intervals, effect sizes, degrees of freedom and *P* value noted<br>*Give P values as exact values whenever suitable.*
☒	☐	For Bayesian analysis, information on the choice of priors and Markov chain Monte Carlo settings
☒	☐	For hierarchical and complex designs, identification of the appropriate level for tests and full reporting of outcomes
☒	☐	Estimates of effect sizes (e.g. Cohen's *d*, Pearson's *r*), indicating how they were calculated
☐	☒	Clearly defined error bars<br>*State explicitly what error bars represent (e.g. SD, SE, CI)*

Our web collection on statistics for biologists may be useful.

Software and code

Policy information about availability of computer code

Data collection	Data were required using PCLAMP 10 recording software. Visual stimuli were generated using Matlab and the Psychophysics Toolbox.
Data analysis	For loose cell-attached recordings, data were analyzed using custom protocols in MATLAB to count the number of spikes. For whole-cell voltage clamp recordings, data were analyzed using PCLAMP 10 software or custom protocols in Matlab to obtain the peak amplitude and total charge transfer of EPSCs and IPSCs.

For manuscripts utilizing custom algorithms or software that are central to the research but not yet described in published literature, software must be made available to editors/reviewers upon request. We strongly encourage code deposition in a community repository (e.g. GitHub). See the Nature Research guidelines for submitting code & software for further information.

Data

Policy information about availability of data

All manuscripts must include a data availability statement. This statement should provide the following information, where applicable:

- Accession codes, unique identifiers, or web links for publicly available datasets
- A list of figures that have associated raw data
- A description of any restrictions on data availability

All relevant data are available upon request.

Field-specific reporting

Please select the best fit for your research. If you are not sure, read the appropriate sections before making your selection.

[x] Life sciences [ ] Behavioural & social sciences [ ] Ecological, evolutionary & environmental sciences

For a reference copy of the document with all sections, see nature.com/authors/policies/ReportingSummary-flat.pdf

Life sciences study design

All studies must disclose on these points even when the disclosure is negative.

Sample size	Final sample size was calculated based on preliminary data to have a power of test stronger than 0.8. Custom Matlab function was used to implement the calculation.
Data exclusions	Six cycles of grating stimuli were present to the cells. Responses during the second to the sixth grating cycles were averaged and the responses during the first cycle were discarded to exclude the influence of cell wake-up responses. Small spike-counts of center-only response is difficult to quantify further suppressive contextual modulation effects since the baseline response is too low to begin with. Therefore, for On or Off spiking datasets, cells with < 10 spike/trial during center-only stimulus were discarded. For PSC datasets, recordings with series resistances > 25 MΩ were discarded to ensure the whole-cell recording quality.
Replication	Key experimental results were found to replicate across cells and mice, and can be reproduced after months.
Randomization	Mice of the same genotype were randomly picked for the experiments. Different visual stimulus conditions were presented in a pseudorandomized sequence generated by Matlab.
Blinding	The investigator was blinded during recording since different stimulus conditions were pseudorandomly generated by Matlab. For data analysis, results were processed using the same Matlab codes for different groups.

Reporting for specific materials, systems and methods

Materials & experimental systems

n/a	Involved in the study
[x]	[ ] Unique biological materials
[x]	[ ] Antibodies
[x]	[ ] Eukaryotic cell lines
[x]	[ ] Palaeontology
[ ]	[x] Animals and other organisms
[x]	[ ] Human research participants

Methods

n/a	Involved in the study
[x]	[ ] ChIP-seq
[x]	[ ] Flow cytometry
[x]	[ ] MRI-based neuroimaging

Animals and other organisms

Policy information about studies involving animals; ARRIVE guidelines recommended for reporting animal research

Laboratory animals	The Gabra2flox/flox mouse line was a generous gift from Dr. Uwe Rudolph at Harvard Medical School. Vgatflox/flox mice (Slc32a1<tm1Lowl>/J), Chat-IRES-Cre mice (129S6-Chattm2(cre)Lowl/J) and floxed tdTomato mice (129S6-Gt(ROSA)26Sortm9(CAG-tdTomato)Hze/J) were acquired from the Jackson Laboratory. Drd4–GFP mice were originally developed by MMRRC (http://www.mmrrc.org/strains/231/0231.html) in the Swiss Webster background, and were subsequently backcrossed to C57BL/6 background. All strains were backcrossed to the C57BL/6 background in our laboratory, and crossed to each other to create the lines used in this study. Mice of ages P22-P68 of either sex were used.
Wild animals	This study did not involve wild animals.
Field-collected samples	This study did not involve field-collected samples.
