# Supplementary material for: Neural mechanisms of contextual modulation in the retinal direction selective circuit
Source: Nat Commun. 2019 Jun 3;10:2431. doi: 10.1038/s41467-019-10268-z (PMC6547848; doi:10.1038/s41467-019-10268-z)
Supplement: Supplementary file 3 — Description of Additional Supplementary Information [file 41467_2019_10268_MOESM3_ESM.pdf]

Description of Additional Supplementary Files

File Name: Supplementary Movie 1
Description: Visual stimuli used in this study.
